# Linking a Gene Cluster to Atranorin, a Major Cortical Substance of Lichens, through Genetic Dereplication and Heterologous Expression

**DOI:** 10.1128/mBio.01111-21

**Published:** 2021-06-22

**Authors:** Wonyong Kim, Rundong Liu, Sunmin Woo, Kyo Bin Kang, Hyun Park, Young Hyun Yu, Hyung-Ho Ha, Seung-Yoon Oh, Ji Ho Yang, Hangun Kim, Sung-Hwan Yun, Jae-Seoun Hur

**Affiliations:** a Korean Lichen Research Institute, Sunchon National University, Suncheon, South Korea; b Research Institute of Pharmaceutical Sciences, College of Pharmacy, Sookmyung Women's University, Seoul, South Korea; c Division of Biotechnology, College of Life Sciences and Biotechnology, Korea University, Seoul, South Korea; d College of Pharmacy, Sunchon National University, Suncheon, South Korea; e Research Institute of Life and Pharmaceutical Sciences, Sunchon National University, Suncheon, South Korea; f Department of Biology and Chemistry, Changwon National University, Changwon, South Korea; g Department of Medical Sciences, Soonchunhyang University, Asan, South Korea; Cornell University

**Keywords:** *Cladonia*, lichen, atranorin, polyketides, secondary metabolism

## Abstract

The depside and depsidone series compounds of polyketide origin accumulate in the cortical or medullary layers of lichen thalli. Despite the taxonomic and ecological significance of lichen chemistry and its pharmaceutical potentials, there has been no single piece of genetic evidence linking biosynthetic genes to lichen substances. Thus, we systematically analyzed lichen polyketide synthases (PKSs) for categorization and identification of the biosynthetic gene cluster (BGC) involved in depside/depsidone production. Our in-depth analysis of the interspecies PKS diversity in the genus *Cladonia* and a related Antarctic lichen, Stereocaulon alpinum, identified 45 BGC families, linking lichen PKSs to 15 previously characterized PKSs in nonlichenized fungi. Among these, we identified highly syntenic BGCs found exclusively in lichens producing atranorin (a depside). Heterologous expression of the putative atranorin PKS gene (coined *atr1*) yielded 4-*O*-demethylbarbatic acid, found in many lichens as a precursor compound, indicating an intermolecular cross-linking activity of Atr1 for depside formation. Subsequent introductions of tailoring enzymes into the heterologous host yielded atranorin, one of the most common cortical substances of macrolichens. Phylogenetic analysis of fungal PKS revealed that the Atr1 is in a novel PKS clade that included two conserved lichen-specific PKS families likely involved in biosynthesis of depsides and depsidones. Here, we provide a comprehensive catalog of PKS families of the genus *Cladonia* and functionally characterize a biosynthetic gene cluster from lichens, establishing a cornerstone for studying the genetics and chemical evolution of diverse lichen substances.

## INTRODUCTION

Lichen-forming fungi (LFF) live in symbiosis with photosynthetic partners, green algae or cyanobacteria—sometimes with both—and LFF are currently estimated to comprise about 20% of all known fungi (1). Lichen symbiosis is one of the most successful mutualisms that enable these organisms to adapt to extremely harsh habitats. A conglutinated, often pigmented, cortical layer made up of dense fungal hyphae provides photosynthetic partners with mechanical stabilization, and algal cells enveloped by hyphae from the medulla provide nutrients to LFF by means of photosynthesis. Secondary metabolites (SMs) of polyketide origin, namely anthraquinones, depsides, depsidones, and dibenzofurans (such as usnic acid), accumulate in cortical or medullary layers of lichen thalli (2). The ecological roles of these lichen SMs are largely unknown, but some studies provide evidence that cortical substances have contributed to habitat expansion (3) and defense from herbivore attacks (4).

Depside and depsidone series compounds are widespread in lichens as cortical and medullary substances, many of which are exclusively found in lichens (2). Lichen depsides are formed by dimerization of either orsellinic acid or 3-methylorsellinic acid (3MOA), and subsequent oxidation of depsides affords tricyclic scaffolds for depsidones. These compounds further undergo different combinations of modification within orsellinic acid and 3MOA moieties, such as alkylation, chlorination, hydroxylation, and *O*-methylation, yielding hundreds of structurally diverse compounds (5, 6). For this, depside/depsidone biosynthetic pathways in lichens could be a model system for studying the substrate specificity of biosynthetic enzymes resulting in chemodiversity. Thus far, however, ascribing specific biosynthetic genes to cortical or medullary substance has been slow in lichens, due to a paucity of genetic information on LFF and lack of molecular tools for manipulating LFF recalcitrant to genetic transformation.

Nonreducing iterative type I polyketide synthases (NR-PKSs) are multidomain enzymes and have been grouped into seven (7) or eight (8) major groups by protein sequence similarity and PKS domain architecture. Orsellinic acid and 3MOA are basic scaffolds for many different SMs in bacteria, fungi, and plants and are biosynthesized by NR-PKSs in a filamentous fungus, Aspergillus nidulans (9, 10), yet only predictions have been made for two NR-PKSs to be responsible for the biosynthesis of the basic units in lichens (11, 12). Atranorin (a 3MOA-derived depside) and usnic acid (a dibenzofuran) are the most common cortical substances of macrolichens and have attracted great attention because of their taxonomic (13, 14), ecological (15), and pharmaceutical (16–19) importance. Several studies have ascribed an NR-PKS to usnic acid with high likelihood (20–22). However, the effort was unsuccessful for heterologous expression of the putative usnic acid PKS gene in Aspergillus oryzae (23), and thus its precise function remains to be determined.

In recent years, SM research has benefited extensively from genome mining approaches in bacteria, fungi, and plants (24–28). We have sequenced eight lichen genomes, including two Antarctic lichens Cladonia borealis and Stereocaulon alpinum, and a foliose *Parmelia* sp. strain, KoLRI021559 (*Parmelia* cf. *squarrosa*), which are reported in this study. Cladoniaceae is one of the largest families of LFF and closely related to Stereocaulaceae (29). Historically, chemotaxonomy has been used as a polyphasic approach to resolve and delimit species boundaries in Cladoniaceae (30, 31), and the genus *Cladonia* has been a model for studying PKS genes for the biosynthesis of cortical and medullary substances (11, 12, 32, 33). Here, we investigated biosynthetic gene cluster (BGC) diversity in six *Cladonia* spp. representing four subsections of the genus and a related lichen, *S. alpinum*, to provide insight into the metabolic potentials of the genus *Cladonia* and to identify BGCs involved in cortical or medullary substances by categorizing PKS genes with a homologous relationship. Also, we established a novel heterologous expression system, in that we reconstructed the biosynthetic pathway for atranorin, a major cortical substance found in nearly every family within the Lecanorales (5), the largest and most diverse order of the class Lecanoromycetes.

## RESULTS

### Metabolic potentials of lichens.

**FIG 1:**
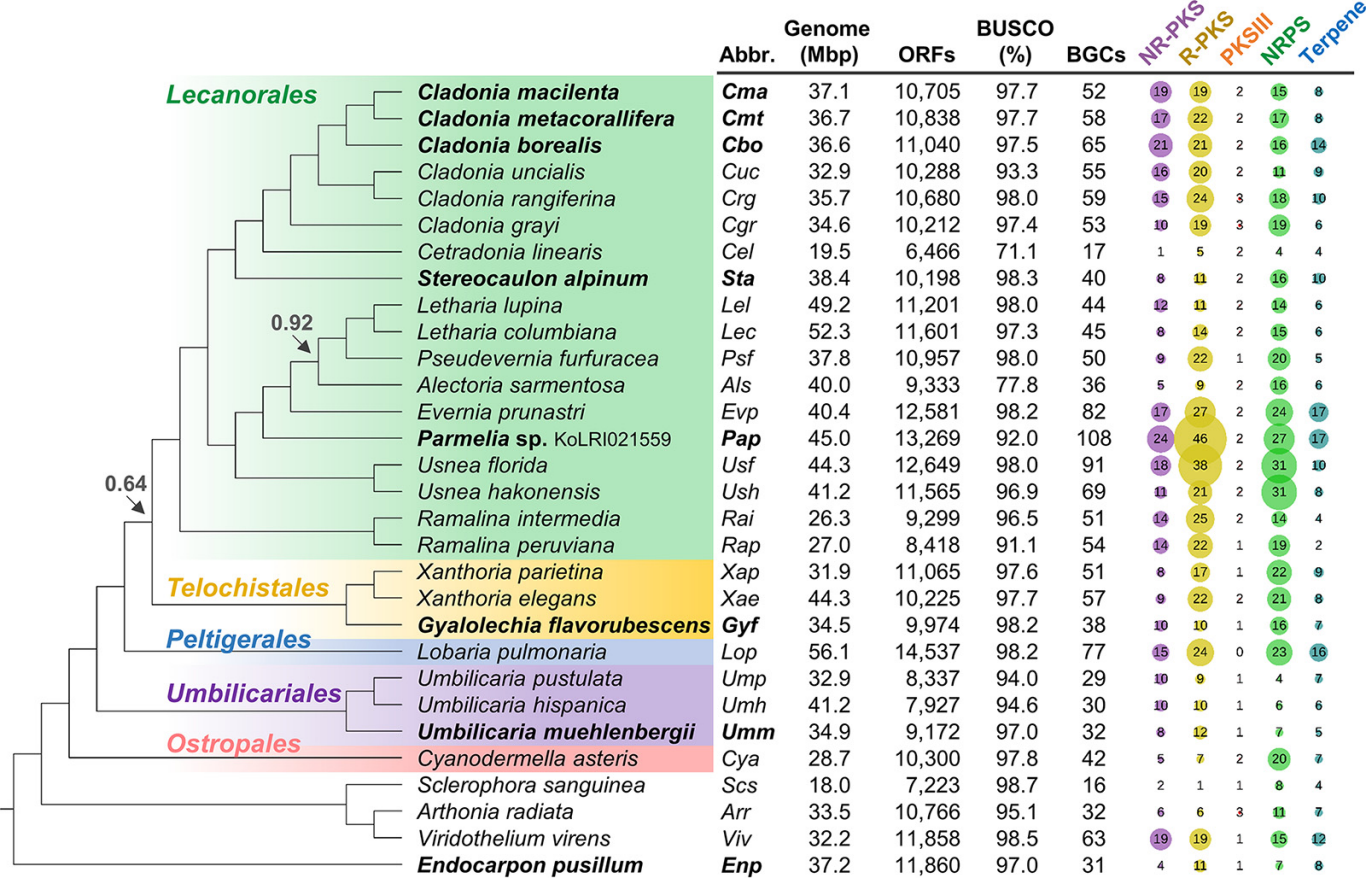
Genome-encoded metabolic potentials of lichens. A coalescent-based species tree is reconstructed for 29 lichen-forming fungi and Cyanodermella asteris (a plant endophyte). The tree is rooted to Endocarpon pusillum. All local posterior probabilities for nodal supports are higher than 0.98, except those marked by arrows. The five orders of the Lecanoromycetes are highlighted with different colors, and lichen genomes sequenced by the Korean Lichen Research Institute are marked in boldface. BUSCO describes the completeness of the genome assemblies. The numbers of biosynthetic gene clusters (BGCs) are predicted by antiSMASH, and bubble plots indicate the relative abundance of core biosynthetic enzyme categories. NR-PKS, nonreducing type I PKS; R-PKS, reducing type I PKS (including PKS-NRPS hybrid enzymes); PKSIII, type III PKS; NRPS, nonribosomal peptide synthetase (including NRPS-like enzymes); and Terpene, biosynthetic enzymes related to terpenoid production.

To examine evolutionary relationships of LFF and compare their genome-encoded metabolic potentials, a coalescent-based tree of 393 single-copy orthologous genes was reconstructed, using eight genomes that we have sequenced and 22 genome assemblies available in the NCBI database and JGI website as of September 2020. A majority of the sequenced species (26 out of 30) belong to the Lecanoromycetes, and four LFF were placed outside the Lecanoromycetes: Arthonia radiata (Arthoniomycetes), Endocarpon pusillum (Eurotiomycetes), Sclerophora sanguinea (Coniocybomycetes), and Viridothelium virens (Dothideomycetes). Barring the genomes of Alectoria sarmentosa and Cetradonia linearis, completeness of the genomes assessed by BUSCO analysis was greater than 91% (Fig. 1). An endangered lichen, *Ce. linearis* (34), and *Sc. sanguinea* had compact genomes shorter than 20 Mbp (Fig. 1). Except for these two species, all genomes were 26.3 to 56.1 Mbp in size, containing 7,927 to 14,537 open reading frames (ORFs) (Fig. 1). Genome-encoded metabolic potentials of the 30 species were investigated by mining the genomes for BGCs using antiSMASH (35). Metabolic potentials of the sequenced LFF were highly variable, with BGC counts of 16 to 108 (Fig. 1); *Parmelia* sp. strain KoLRI021559 showed the greatest BGC diversity, whereas the genomes of *Ce. linearis* and *Sc. sanguinea* carried the smallest sets of BGCs, which may be attributable to their contracted genome size. Notably, the six *Cladonia* genomes possess 52 to 65 BGCs and encode similar numbers of PKSs: an average of 16 NR-PKSs and 20 reducing-type PKSs (R-PKSs) (Fig. 1). The six *Cladonia* spp. produce unique sets of SMs of polyketide origin (see Table S1 and Fig. S1 in the supplemental material), which make them ideal for studying interspecies PKS gene diversity and identifying BGCs responsible for biosynthesis of cortical or medullary substances of lichens through comparative analysis. Full details on genome statistics and BGC information for the 30 sequenced species are provided in Data Set S1 in the supplemental material.

10.1128/mBio.01111-21.2 FIG S1 Chemical structures of cortical and medullary substances of lichens. Download FIG S1, PDF file, 0.2 MB. Copyright © 2021 Kim et al. 2021 Kim et al. https://creativecommons.org/licenses/by/4.0/ This content is distributed under the terms of the Creative Commons Attribution 4.0 International license.

10.1128/mBio.01111-21.7 TABLE S1 Lichen substances reported in the 30 genome-sequenced species. Download Table S1, DOCX file, 0.02 MB. Copyright © 2021 Kim et al. 2021 Kim et al. https://creativecommons.org/licenses/by/4.0/ This content is distributed under the terms of the Creative Commons Attribution 4.0 International license.

10.1128/mBio.01111-21.9 DATA SET S1 Genome statistics and biosynthetic gene clusters in the sequenced lichen species. Download Data Set S1, XLSX file, 0.02 MB. Copyright © 2021 Kim et al. 2021 Kim et al. https://creativecommons.org/licenses/by/4.0/ This content is distributed under the terms of the Creative Commons Attribution 4.0 International license.

### Genetic dereplication of *Cladonia* PKSs.

**FIG 2:**
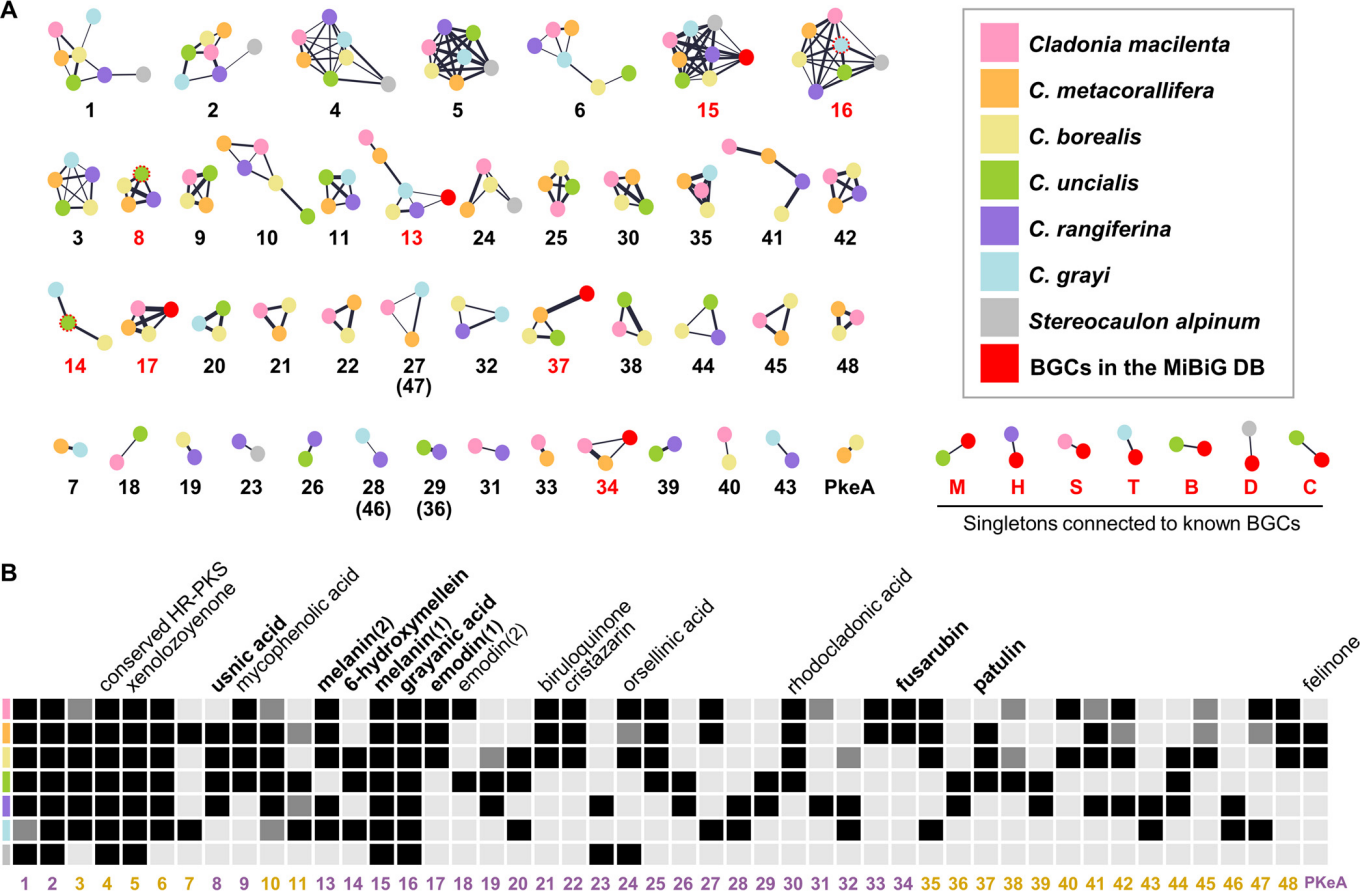
Polyketide BGC diversity in *Cladonia* species. (A) Forty-five gene cluster families (GCFs) were identified by gene cluster network analysis in the six *Cladonia* spp. and Stereocaulon alpinum. GCFs are labeled with their associated PKS families. Numbers in red indicate PKS families connected to characterized BGCs deposited in the MIBiG database. Nodes indicate BGCs and are color coded by species (see the inset). Nodes enclosed by a red dashed circle in the PKS8, PKS14, and PKS16 subnetworks are lichen BGCs in the MIBiG database. Edge width was drawn proportional to squared similarity between a pair of BGCs. Singleton BGCs that connected to known BGCs are labeled as follows: M, monascorubrin BGC; H, hypothemycin BGC; S, sorbicillin BGC; T, terreic acid BGC; B, betaenone BGC; D, depudecin BGC; C, curvupallides BGC. (B) Phyletic distribution of PKS families evidenced by gene cluster network analysis. Numbers in violet and ochre indicate NR-PKS and R-PKS families, respectively. Black squares indicate the presence of PKS families, and gray squares indicate pseudogenes or partial genes due to incomplete gene annotation. Signature secondary metabolites linked to *Cladonia* PKS families by genetic dereplication are marked in boldface.

To search for PKS genes involved in biosynthesis of lichen polyketides, namely depside and depsidone series compounds, we focused on 226 BGCs harboring at least one PKS in the six *Cladonia* spp. plus a related Antarctic lichen, *S. alpinum*, from which a total of 242 PKSs were identified. We first conducted clustering analysis of the conserved ketosynthase (KS) domains of each PKS and identified a number of clusters that are suggestive of homologous relationships (see Fig. S2 in the supplemental material). Twelve PKS families described in an earlier study on the Cladonia chlorophaea species complex (33) were also detected in our clustering analysis, indicating that a significant proportion of PKS genes are conserved in the genus *Cladonia*. However, little is known about their products. Therefore, we linked BGCs to known compounds in nonlichenized fungi, using the Big-SCAPE program, which maps BGC diversity onto sequence similarity networks (36). The network analysis was used to graphically summarize three attributes of BGCs: (i) PKS families associated with the network (numbers), (ii) species distribution across the network (nodes), and (iii) degrees of similarity between pairs of BGCs (edges) (Fig. 2A). As depicted by the gene cluster network, the BGCs harboring at least one PKS gene were grouped into 45 gene cluster families (GCFs). Eight GCFs and seven species-specific BGCs (labeled in red) were coupled to previously characterized BGCs deposited in the MIBiG database (37). Figure 2B summarizes the phyletic distribution of *Cladonia* PKS families, denoting signature SMs linked to *Cladonia* PKS families. Full annotations of the PKS families linked to known compounds are provided in Text S1 in the supplemental material.

10.1128/mBio.01111-21.1 TEXT S1 Supplemental materials and methods. Download Text S1, DOCX file, 0.2 MB. Copyright © 2021 Kim et al. 2021 Kim et al. https://creativecommons.org/licenses/by/4.0/ This content is distributed under the terms of the Creative Commons Attribution 4.0 International license.

10.1128/mBio.01111-21.3 FIG S2 Overview of the similarity of PKS families. Download FIG S2, PDF file, 0.4 MB. Copyright © 2021 Kim et al. 2021 Kim et al. https://creativecommons.org/licenses/by/4.0/ This content is distributed under the terms of the Creative Commons Attribution 4.0 International license.

### Identification of a putative atranorin BGC.

**FIG 3:**
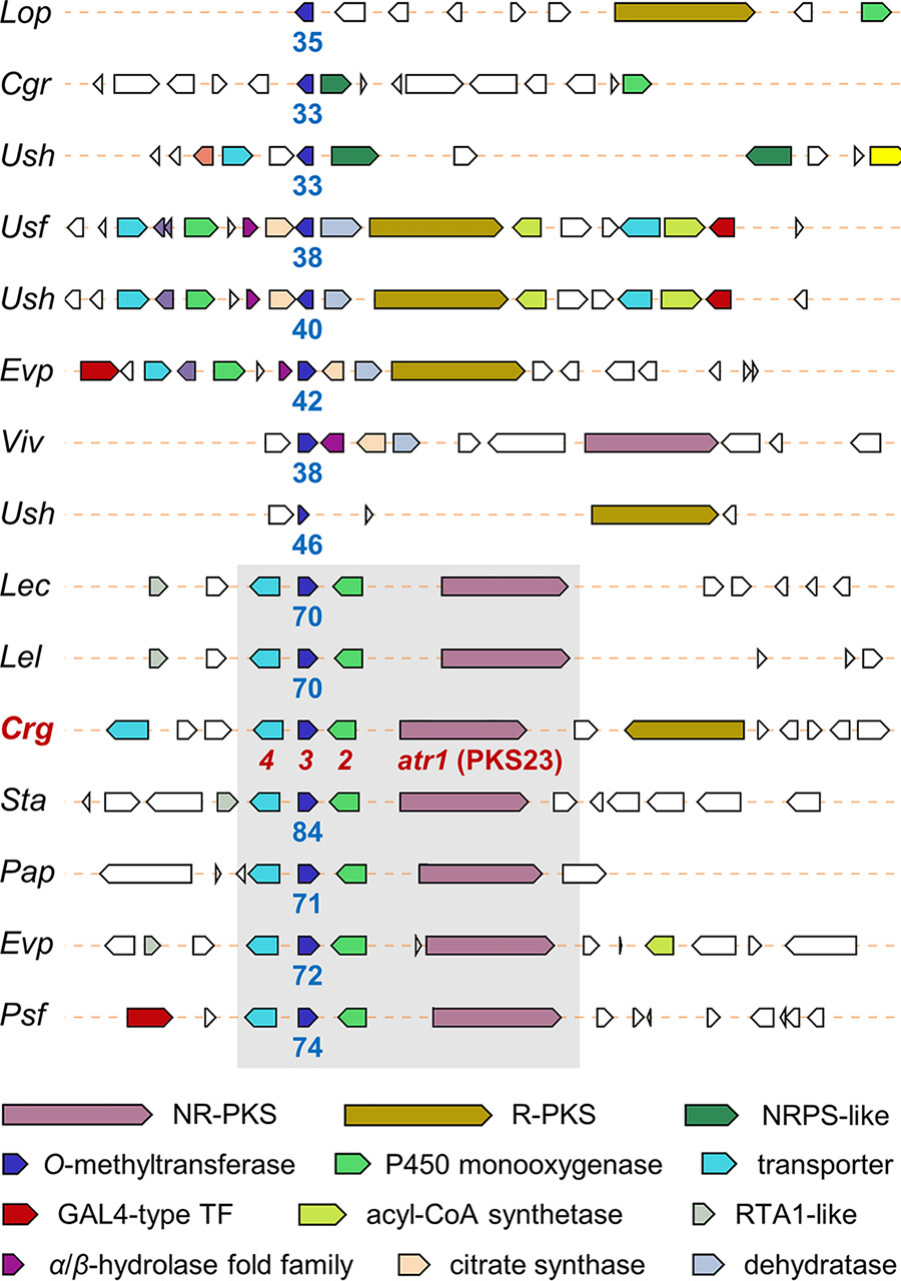
Organization of putative atranorin BGCs. Fifteen BGCs, including an *O*-methyltransferase that shows significant hits to the one in the PKS23 BGC in *C. rangiferina* (*Crg*; labeled in red) are identified from 1,527 BGCs detected in the 30 genomes, using the CORASON analysis pipeline (36). Lichen species that harbor the BGCs are labeled with their abbreviated names, as in Fig. 1. Numbers in blue indicate the percentage of protein sequence identity of homologous OMTs to the one in *C. rangiferina*. Among the 15 BGCs, seven BGCs harbor four syntenic genes (shaded box): *atr1* (PKS23), *atr2*, *atr3*, and *atr4*. Genes related to secondary metabolism are color coded, based on their predicted function. NR-PKS, nonreducing type I PKS; R-PKS, reducing type I PRS; NRPS, nonribosomal peptide synthetase; TF, transcription factor; RTA, resistance to 7-aminocholesterol.

The gene cluster network analysis of the six *Cladonia* spp. and *S. alpinum* revealed that the PKS23 family among the 45 GCFs may be involved in biosynthesis of atranorin, the major cortical substance of diverse macrolichens, as it was the sole GCF shared by the two atranorin producers, *C. rangiferina* and *S. alpinum* (Fig. 2). Atranorin is unique in its structure having a methoxycarbonyl group within the 3MOA moiety, which is rarely found in other depside and depsidone series compounds. A BLAST search with an *O*-methyltransferase (OMT) in the PKS23 BGC in *C. rangiferina* showed 37% protein sequence identity to Trt5 (UniProtKB accession no. Q0C8A3), an OMT that mediates the formation of a methoxycarbonyl group within the 3,5-dimethylorsellinic acid moiety in A. terreus during the biosynthesis of terretonin (a meroterpenoid) (38) (Table 1). To examine the presence of conserved PKS23 BGCs in other lichens that produce atranorin, we adopted the CORASON pipeline (36), which is useful for studying conservation and variation of BGCs within and across GCFs. The protein sequence of the OMT in the PKS23 BGC in *C. rangiferina* was queried against a total of 1,527 BGCs detected in the 30 genomes. We identified 15 BGCs, each harboring an OMT that showed a significant hit (>33% protein sequence identity) (Fig. 3). Among the 15 BGCs, seven BGCs contained four syntenic core genes: the PKS23 gene (coined *atr1*), a cytochrome P450 monooxygenase gene (*atr2*), an OMT gene (*atr3*), and a transporter gene (*atr4*) (Fig. 3 and Table 1). These syntenic BGCs were exclusively found in the atranorin-producing lichens among the 30 sequenced species (Table S1).

**TABLE 1:** The PKS23 biosynthetic gene cluster in Cladonia rangiferina

ORF^*a*^	Size (aa)	BLASTP homolog^*b*^	Identity (%)	Conserved domain	E value
06811	744	MFS monosaccharide transporter	71	Sugar transporter (Pfam00083)	1e−105
06812	306	Export control protein CHS7-like	68	Chitin synthase III catalytic subunit (Pfam12271)	1e−131
06813	495	TFIIH complex p47 subunit	58	TFIIH complex subunit Ssl1-like (Pfam04056)	1e−82
**06814**	549	MFS multidrug transporter (***atr4***)	50	Major facilitator superfamily (Pfam07690)	9e−31
**06815**	346	Trt5, *O*-methyltransferase (***atr3***)	37	SAM-dependent methyltransferase (Pfam08241)	1e−04
**06816**	508	Cytochrome P450 monooxygenase (***atr2***)	43	Cytochrome P450 (Pfam00067)	3e−57
**06817**	2,513	Nonreducing polyketide synthase (***atr1***)	44	SAT-KS-AT-PT-ACP-MT-TE^*c*^	
06818	472	Hypothetical protein	33	Phosphotransferase enzyme family (Pfam01636)	4e−08
06819	2,377	Reducing polyketide synthase (R-PKS)	40	KS-AT-DH-ER-KR-ACP^*c*^	
06820	184	Hypothetical protein	40	Domain of unknown function, DUF3237 (Pfam11578)	5e−22
06821	301	Hypothetical protein	34	Domain of unknown function, DUF2306 (Pfam10067)	2e−09
06822	236	Hypothetical protein	28	Not detected	
06823	356	Hypothetical protein	32	Not detected	
06824	565	Putative dimethylaniline monooxygenase	41	Flavin-binding monooxygenase-like (Pfam00743)	3e−14

a ORF, *C. rangiferina* open reading frame. The syntenic core genes are highlighted in boldface.

b For BLAST searches, the NCBI nonredundant protein sequence database for four Aspergillus species was used: Aspergillus fumigatus (taxid 746128), A. nidulans (taxid 162425), A. niger (taxid 5061), and A. terreus (taxid 33178).

c For PKS domain architecture, see Data Set S2 in the supplemental material.

10.1128/mBio.01111-21.10 DATA SET S2 NR-PKSs characterized in nonlichenized fungi and NR-PKSs found in the six *Cladonia* spp. and *Stereocaulon alpinum*. Download Data Set S2, XLSX file, 0.5 MB. Copyright © 2021 Kim et al. 2021 Kim et al. https://creativecommons.org/licenses/by/4.0/ This content is distributed under the terms of the Creative Commons Attribution 4.0 International license.

### Reconstruction of the atranorin biosynthetic pathway.

**FIG 4:**
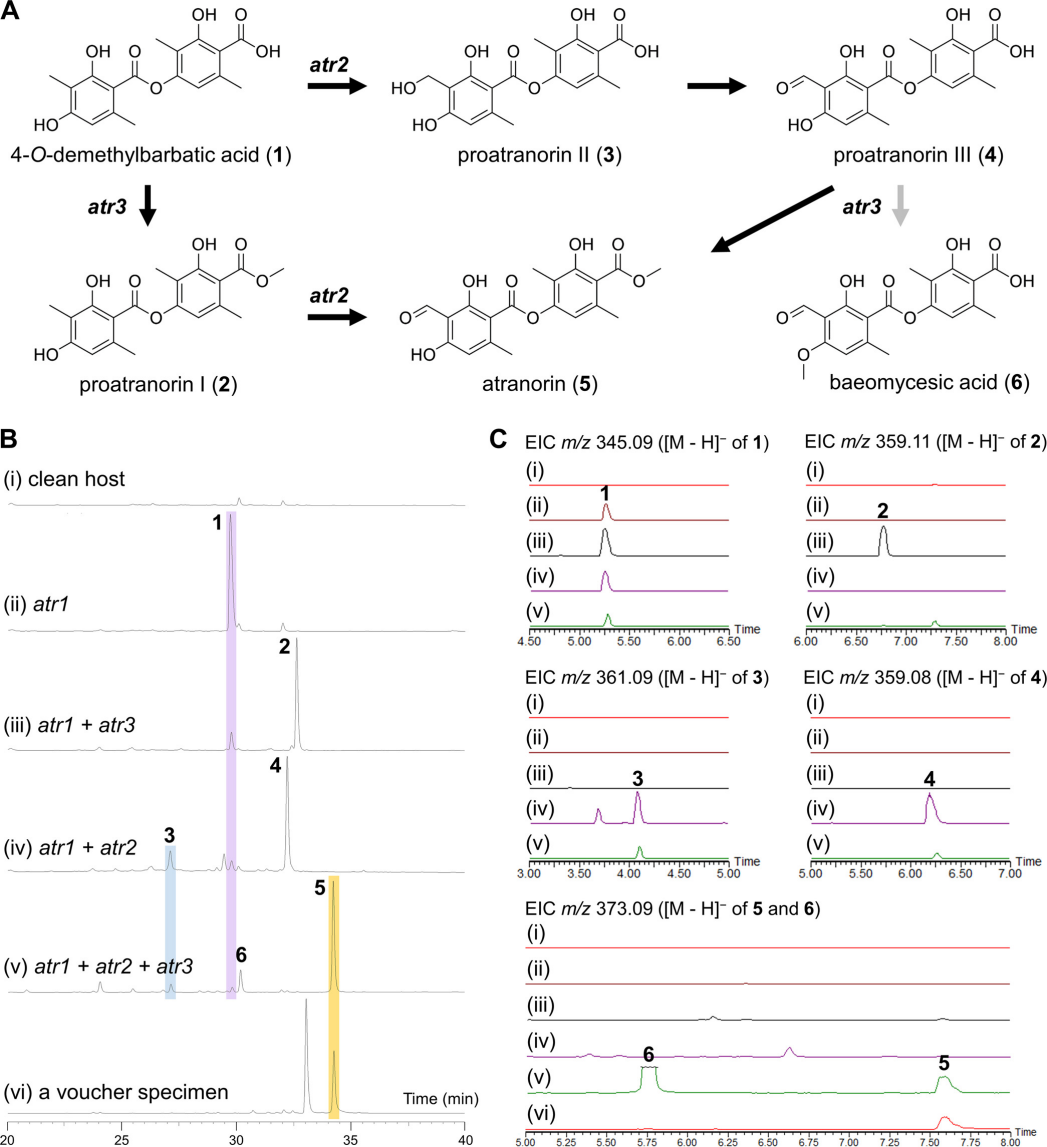
Functional validation of the atranorin BGC. (A) A proposed biosynthetic pathway for atranorin. (B) HPLC profiles of culture extracts of a “clean host” expressing different sets of biosynthetic genes of the atranorin BGC: with no introduced gene (i), with *atr1* only (ii), with *atr1* and *atr3* (iii), with *atr1* and *atr2* (iv), and with *atr1*, *atr2*, and *atr3* (v). The acetone extract of an authentic voucher specimen for the genome-sequenced Stereocaulon alpinum contains atranorin (compound 5) and lobaric acid (*t*_R_ = 33.1 min) (vi). (C) Extracted-ion chromatograms (EIC) with the indicated *m*/*z* values for compounds 1 to 6.

Despite arduous efforts made for heterologous expression of lichen PKS genes in well-established heterologous hosts, such as A. nidulans and A. oryzae, the attempts were unsuccessful for yet unknown reasons (11, 23). Thus, we set out to establish a new heterologous expression system, using a plant-pathogenic fungus, Ascochyta rabiei (Dothideomycetes). On the basis of the chemical structure and the predicted functions of three biosynthetic genes in putative atranorin BGCs, a polyketide pathway for atranorin can be envisioned (Fig. 4A). To investigate the roles of individual genes, we first generated a “clean host” that showed no appreciable metabolite production by removing the BGC for biosynthesis of solanapyrones from the wild-type *As. rabiei* (39, 40) (Fig. 4B). Then, we introduced the *atr1* gene cloned from the *S. alpinum* genomic DNA (gDNA) into the “clean host.” Expression and correct intron splicing of the *atr1* were confirmed by reverse transcription-PCR (RT-PCR) analyses (see Fig. S3 in the supplemental material). The liquid chromatography-tandem mass spectrometry (LC-MS/MS) and nuclear magnetic resonance (NMR) analyses indicated a known lichen depside, 4-*O*-demethylbarbatic acid (compound 1), was produced by a strain expressing the *atr1* gene (Fig. 4B and C; see Fig. S4A in the supplemental material), suggesting that two 3MOA units were joined into a depside by Atr1 itself.

10.1128/mBio.01111-21.4 FIG S3 Verification of heterologous expression of PKS23. Download FIG S3, PDF file, 0.1 MB. Copyright © 2021 Kim et al. 2021 Kim et al. https://creativecommons.org/licenses/by/4.0/ This content is distributed under the terms of the Creative Commons Attribution 4.0 International license.

10.1128/mBio.01111-21.5 FIG S4 NMR and LC-MS/MS spectroscopic analyses. Download FIG S4, PDF file, 0.7 MB. Copyright © 2021 Kim et al. 2021 Kim et al. https://creativecommons.org/licenses/by/4.0/ This content is distributed under the terms of the Creative Commons Attribution 4.0 International license.

As the biosynthesis of atranorin from compound 1 requires oxidation of a methyl group and methylation of the carboxyl group within each of the two 3MOA units (Fig. 4A), we individually introduced the *atr2* and *atr3* genes into the *atr1*-expressing strain. Coexpression of the *atr1* gene with the *atr3* gene yielded a 7′-*O*-methylated analogue of compound 1, proatranorin I (compound 2), indicating that Atr3 is indeed a carboxyl methylase, as is Trt5 in A. terreus (Fig. 4B and C; Fig. S4B). The LC-MS/MS analysis showed that coexpression of *atr1* with *atr2* yielded compounds 3 and 4, which were annotated as a hydroxylated analogue of compound 1 (proatranorin II) and a further oxidized aldehyde (proatranorin III), respectively (Fig. 4B and C; Fig. S4C). NMR analysis of the isolated proatranorin III (compound 4) confirmed that the oxidation by Atr2 occurs at C-9 of compound 1 (Fig. S4D). Finally, we generated a strain expressing the three genes *atr1*, *atr2*, and *atr3*. The production of atranorin (compound 5) was confirmed by MS/MS spectral matching with the reference spectrum of atranorin in the Lichen Database (41) (Fig. S4E). It was also confirmed by comparison of the chemical profile with an authentic voucher specimen for the sequenced *S. alpinum*, which produces atranorin (compound 5) and lobaric acid, as a cortical substance and medullary substance, respectively (Fig. 4B and C). Intriguingly, MS/MS spectral matching of compound 6 (Fig. S4E) identified it as another lichen depside, baeomycesic acid, which has not been reported from *S. alpinum* in nature, suggesting that Atr3 exhibits relaxed substrate specificity in the heterologous host.

### A novel phylogenetic clade of PKS responsible for biosynthesis of lichen substances.

**FIG 5:**
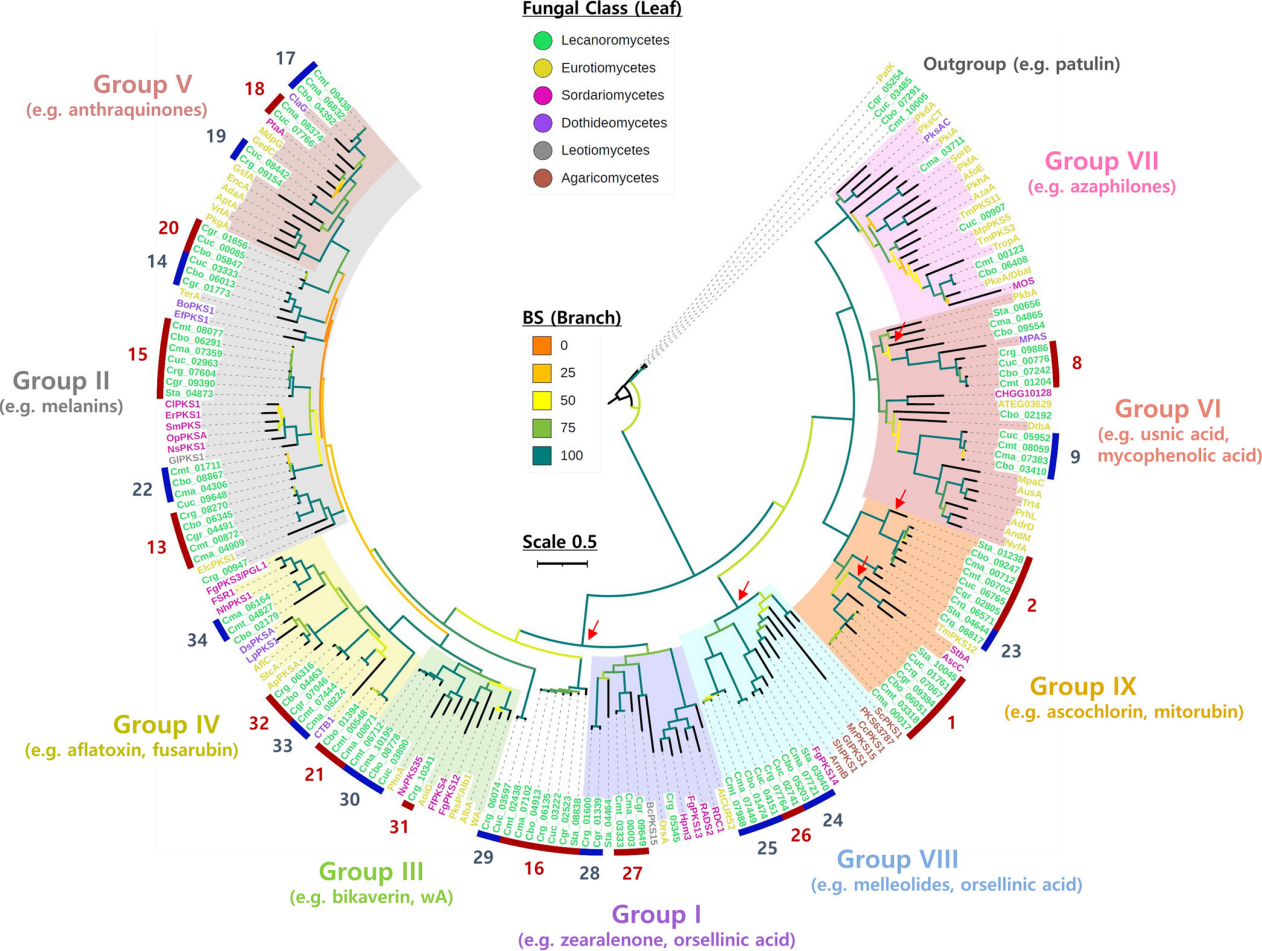
The ninth clade of fungal nonreducing PKS genes. A maximum likelihood tree of 103 NR-PKSs in the six *Cladonia* spp. and *Stereocaulon alpinum* and 82 NR-PKSs linked to known compounds in nonlichenized fungi was reconstructed using concatenated sequences of ketosynthase and product template domains. The outgroup was set to 6-methylsalicylic acid synthases (6MSAS) found in *Cladonia* spp. and the 6MSAS for the patulin biosynthesis in Penicillium expansum. Branches are color coded, based on bootstrap (BS) values. The scale represents 0.5 amino acid sequence substitution per site. Blue and red strips with associated numbers at the outermost region indicate PKS families identified by the gene cluster network analysis (Fig. 2). Phylogenetic clades representing nine NR-PKS groups (groups I to IX) are shaded with different colors. The NR-PKS groups are supported by BS values greater than 75, except for group II. Note that the newly identified NR-PKS group (group IX) includes the PKS1, PKS2, and PKS23 families, candidate PKSs for cortical and medullary substances of lichens. Red arrows indicate polyphyletic losses of the *C*-methyltransferase domain of NR-PKSs.

To study the evolutionary relationships of fungal NR-PKS and the Atr1 responsible for atranorin production, we reconstructed a phylogenetic tree of the concatenated sequences of conserved KS and product template (PT) domains of 103 NR-PKSs found in the six *Cladonia* spp. and *S. alpinum* and 82 NR-PKSs that have been linked to known compounds in nonlichenized fungi (see Data Set S2 in the supplemental material). Fungal NR-PKSs were hitherto largely classified into eight groups (groups I to VIII) in previous phylogenetic analyses (7, 8, 42–44). Here, we identified a novel NR-PKS group (group IX) supported by a 100% bootstrap, which contained the PKS23 family as well as the PKS1 and PKS2 families (Fig. 5). The PKS1 family was previously proposed to biosynthesize 3MOA-derived lichen substances in *C. rangiferina* (12). For lichen NR-PKSs in group IX, all but the PKS2 in *S. alpinum* possessed a *C*-methyltransferase (cMT) domain involved in the methylation of polyketide intermediates (Data Set S2). Intriguingly, the newly identified group IX included three previously characterized NR-PKSs, AscC (45), StbA (46), and TmPKS12 (47), in nonlichenized fungi (Fig. 5). These NR-PKSs lack the cMT domain and are known to produce orsellinic acids (45–47) (Data Set S2). Moreover, it is noteworthy that the group VIII, basal to group IX, included several NR-PKSs that have been linked to orsellinic acid-derived compounds in mushroom-forming fungi (Agaricomycetes) (48–51) and Fusarium graminearum (52) (Fig. 5; Data Set S2), indicating that the PKS1 and PKS2 families are likely involved in biosynthesis of lichen substances derived from a methylated form of orsellinic acid, as with the PKS23 family.

## DISCUSSION

Many lichen SMs are of ecological and pharmaceutical importance; however, lack of genetic tools for studying LFF has hindered identification of BGCs and industrial applications of lichen substances. Our in-depth genomic analysis of the interspecies BGC diversity in the genus *Cladonia* and heterologous expression of the atranorin BGC identified the complete biosynthetic pathway for atranorin, the major cortical substance of lichens, setting the first precedent for successful genome mining approaches for lichen SMs.

### Biosynthesis of cortical and medullary substances in lichens.

Atranorin and usnic acid tend to be found mutually exclusively in lichens, albeit some taxa are known to produce both as cortical substances (e.g., Evernia prunastri) (53). This is true especially for the genus *Cladonia*; the presence of either usnic acid or atranorin in cortical layers is often used as a key trait for the chemotaxonomy (30). We found that *C. rangiferina* and *Parmelia* sp. strain KoLRI021559 carry both atranorin and usnic acid BGCs in the genomes; however, it was not usnic acid but atranorin that was found in their cortex (see Fig. S5A and S5B in the supplemental material), supporting the previous hypothesis that usnic acid has been replaced with atranorin as a major cortical substance independently in different lineages of LFF, especially in parmelioid lichens (13, 21). The PKS1 and PKS23 families are identical in gene structure, with 4 introns at conserved positions. The presence of the PKS1 family in *Sc. sanguinea* outside the Lecanoromycetes and highly syntenic PKS23 BGCs compared to the PKS1 BGCs (Fig. S5C) suggested that the PKS1 family may be ancestral and that the PKS23 family may have arisen from a duplication event in the common ancestor of atranorin-producing lichens, and then was adopted as a major cortical substance in some lineages or purged from the others, shaping the current phyletic distribution of atranorin in the Lecanoromycetes (5). Although the function of the PKS1 remains to be characterized, we speculate that the PKS1 family is involved in biosynthesis of medullary substances derived from 3MOA units, based on the fact that the expression level of a PKS1 homolog in Usnea rubrotincta (denoted UrPKS5) is highly correlated with *in vitro* production of medullary depsidones, such as salazinic acid and norsticitic acid (54). The PKS1 and PKS23 families may be an example of neofunctionalization of duplicate genes that have been functionally diverged to produce spatially distinct metabolites in macrolichens.

10.1128/mBio.01111-21.6 FIG S5 Lichens with atranorin and usnic acid BGCs. Download FIG S5, PDF file, 0.5 MB. Copyright © 2021 Kim et al. 2021 Kim et al. https://creativecommons.org/licenses/by/4.0/ This content is distributed under the terms of the Creative Commons Attribution 4.0 International license.

### Phylogenetic relationship of NR-PKSs for depside and depsidone biosynthesis.

The phylogenetic analysis of fungal NR-PKSs revealed two major clades (groups I to V and groups VI to IX). The ancestral NR-PKS was estimated to have a cMT domain, because this domain is present both in fungal NR-PKS and R-PKS, as well as in bacterial R-PKS (55). The groups I to V seem to have diverged after the loss of the cMT domain (55). Also, we observed that there has been polyphyletic loss of the cMT domain in some lichen NR-PKSs within groups VI and IX and presumably during the rise of group VIII diverging from groups I to V. In nonlichenized fungi, several NR-PKSs within groups I and VIII (two basal clades lacking the cMT domain) exhibit intra- or intermolecular cross-linking activities when the enzymes release polyketide intermediates from the active sites, forming depsides, macrolides, and melleolides, all derived from orsellinic acid (48, 50, 56, 57). These two ancestral clades may include NR-PKSs responsible for biosynthesis of depsides and depsidones derived from orsellinic acid, such as the PKS16 family (sister to group I) for grayanic acid production in Cladonia grayi (11). The discovery of the PKS23 family in group IX with both cross-linking and cMT activities is particularly exciting, as this finding directs future research focus on the ninth clade of NR-PKS to resolve long-standing questions of how lichens form structurally diverse depside and depsidone compounds derived from 3MOA.

### Lichen BGC diversity treasure chests for novel SMs.

The numbers of NR-PKSs found in the six *Cladonia* spp. placed their metabolic repertoire on par with those of four representative Aspergillus spp. that have been models for SM studies in nonlichenized fungi (58). More than two-thirds of NR-PKSs were found to be within groups V to VII in each of the four Aspergillus spp. lacking NR-PKS within group IX, whereas *Cladonia* spp. tended to have diverse NR-PKS groups and possessed 2 to 3 NR-PKSs belonging to group IX (Fig. S5D). Despite huge metabolic potentials encoded in each lichen genome, only a few SMs occur in each lichen thallus as major cortical or medullary substances, implying that most of the BGCs remain silent in nature. Our gene cluster network analysis expanded the knowledge on lichen BGCs connected to known compounds. However, there are still a lot more cryptic BGCs, many of which are species or lineage specific and may be involved in biosynthesis of novel compounds. Given the frequent appearance of unexpected metabolites (not observed in natural lichen thalli) in axenic culture of LFF (59–63), many of these cryptic BGCs appear to be still functional.

### Future perspectives.

As lichen genomes become more and more available, we soon will be able to draw a more comprehensive picture of metabolic diversity and evolutionary fate of lichen BGCs. Comparative analyses of BGC contents between lichens producing structurally related compounds will identify biosynthetic enzymes harnessing structural diversity of cortical and medullary substances in lichens. This information can be applied to combinatorial biosynthesis of lichen-derived compounds with improved pharmacological activities. Our newly established heterologous expression system would help us understand the role of biosynthetic enzymes for well-known lichen substances and enable production of novel SMs encoded by cryptic BGCs in lichens.

## MATERIALS AND METHODS

### Phylogenomic analysis.

To infer the phylogeny of the genome-sequenced 29 LFF and an endophytic fungus Cyanodermella asteris, we pursued a coalescent-based phylogenomics approach. The culture, genome sequencing, and annotation procedures for LFF are described in Text S1. Single-copy ortholog clusters (SCOs) of protein sequences deduced from the 30 annotated genomes were identified using OrthoMCL (v2.0.9) (64) with an inflation factor of 2.5. For each of the 393 SCOs, protein sequences were aligned using MAFFT (v7.310) (65) with the “auto” setting, and the resulting 393 multiple-sequence alignments were trimmed for poorly aligned regions using Gblocks (v0.91b) (66) with the parameter “-b4 = 5.” The RAxML program (v8.2) (67) was used to calculate 100 maximum likelihood trees for each multiple-sequence alignment. To generate coalescent-based trees, we used ASTRAL-III (v5.7.4) (68) with two multilocus bootstrapping options (site-only resampling and gene/site resampling) and with no bootstrapping option. All trees showed the identical topology.

### Biosynthetic gene cluster family analysis.

For BGC identification in the 30 genomes, the genome assembly and annotation files were processed by the antiSMASH program (v5.0+) (35), with the parameter setting “–minimal.” For genetic dereplication of BGCs found in the six *Cladonia* spp. and *S. alpinum*, we used the BiG-SCAPE program (36), with reference to the MIBiG database (v1.4) (37). To analyze BGCs containing at least one iterative type I PKS, we modified a configuration file, “domain_whitelist.txt,” to include only Pfam domains related to the iterative type I PKS (PF00109 and PF02801) and ran the BiG-SCAPE program with optional arguments “–mix” and “–hybrids-off.” Based on the Jaccard index of domain types, domain sequence similarity, and domain adjacency index, the BiG-SCAPE program calculates a similarity matrix between pairwise combinations of clusters, where smaller values indicate greater BGC similarity (36). We evaluated BGC networks using an edge-length cutoff from 0.3 to 0.8 with a step of 0.1 and considered the network using a cutoff value of 0.5 as a representation of GCFs in the six *Cladonia* spp. and *S. alpinum*. Individual networks for PKS families were visualized using a Python package NetworkX (v2.5) (69), in that edge width was weighted by squared similarity between a pair of BGCs calculated by BiG-SCAPE. We used the CORASON program (36), which generates a multilocus, approximately maximum likelihood, phylogenetic tree of BGCs, including PKS families responsible for production of lichen substances. For the PKS23 family, an OMT gene (Crg06815) in *C. rangiferina* was used as the query gene to search for homologous BGCs from a total of 1,527 BGCs detected in the 30 sequenced species. For the PKS1 family, a GAL4-type transcription factor gene (GeneID Crg07068) in *C. rangiferina* was used as the query gene.

### Phylogenetic analysis of fungal NR-PKS.

The KS domain of iterative type I PKS has been considered evolutionarily conserved (55), and thus it can serve as a proxy for the similarity of the entire PKS. We identified a total of 242 PKSs from the genomes of the six *Cladonia* spp. and *S. alpinum*, among which four PKSs (Cgr01615, Cgr03964, Cgr08611, and Cmt10189) (Data Set S2) were missing a KS domain. KS domain sequences were extracted from 238 PKSs using the online tool NaPDoS (70) and aligned using MUSCLE (v3.8.31) (71). For clustering analysis, an all-versus-all similarity matrix for KS domains of 238 PKSs was computed using the AlignBuddy function in the BuddySuite program (72), with an optional argument, “-pi.” A heat map showing the percentage of similarity of KS domains clustered by *k*-means was generated using the R package Superheat (73). For fungal NR-PKS phylogeny, we used concatenated protein sequences of KS and PT domains of 103 NR-PKSs found in the six *Cladonia* spp. and *S. alpinum* and 82 NR-PKSs that have been linked to known compounds in nonlichenized fungi (7, 8, 44) (Data Set S2). We initially identified 106 NR-PKSs in the six *Cladonia* spp. and *S. alpinum*, including seven NR-PKSs whose full sequences cannot be reliably defined from the current genome assembly (likely pseudogenes). Among these partial PKSs, three NR-PKSs (Cbo04702, Cma06590, and Cmt06606) (Data Set S2) lacked either the KS or PT domain and were excluded from the phylogenetic analysis. PT domains of lichen NR-PKSs were identified by aligning with those of previously characterized PKSs (8). A 6-methylsalicylic acid synthase (6MSAS) responsible for the biosynthesis of patulin (UniProtKB accession no. A0A075TRC0) in Penicillium expansum was set to be an outgroup to fungal NR-PKS phylogeny. Also, four 6MSASs found in four *Cladonia* spp. were included in the analysis (Cbo07291, Cgr05254, Cmt10005, and Cuc03485) (Data Set S2). Protein sequences of KS and PT domains were aligned using MAFFT (v7.310) (65) with the “auto” setting, and spurious sequences or poorly aligned regions from each domain were trimmed using the trimAl program (v1.2) (74), with the “gappyout” parameter. The resulting multiple-sequence alignments for KS and PT domains were concatenated with FASconCAT-G (v1.04) (75). From the concatenated sequences, maximum likelihood trees were computed with RAxML (v8.2) (67), using a gamma distribution for substitution rate across sites with the parameter setting “-m PROTGAMMAWAG.” Nodal support was evaluated by 1,000 bootstrap replications. The final tree was rooted to the 6MSAS outgroup and annotated by iTOL (v5.7) (76).

### Generation of a “clean host” from *Ascochyta rabiei*.

A split marker strategy (77) was employed to generate a solanapyrone-negative mutant (“clean host”). A downstream region of the *sol1* gene (1,208 bp) and an upstream region of the *sol3* gene (1,459 bp) were amplified from gDNA of *As. rabiei* isolate AR628 (39), using the L5/L3 and R5/R3 primer pairs, respectively. A hygromycin phosphotransferase gene (*hph*) cassette was amplified from the pCB1004 plasmid (78), using thee primer pair HYG5/HYG3. The L3 and R5 primers have 27-nucleotide (nt)-long overhang sequences complementary to the 3′ and 5′ ends of the *hph* cassette, respectively, such that the amplified *sol1* downstream and *sol3* upstream regions can be fused to the *hph* cassette by overlap extension PCR. Finally, split marker constructs were amplified from the fused construct, using N5/HY-R and YG-F/N3 primer pairs. The split marker constructs were introduced into protoplasts of *As. rabiei* by polyethylene glycol-mediated genetic transformation as described by Hallen-Adams et al. (79). The primers used in the gene replacement are listed in Table S2 in the supplemental material.

10.1128/mBio.01111-21.8 TABLE S2 Primers used in this study. Download Table S2, DOCX file, 0.03 MB. Copyright © 2021 Kim et al. 2021 Kim et al. https://creativecommons.org/licenses/by/4.0/ This content is distributed under the terms of the Creative Commons Attribution 4.0 International license.

### Heterologous expression of the *atr1* and RT-PCR analysis.

For efficient expression of foreign genes in the “clean host,” we constructed expression vectors that carry either the *sol1* gene promoter (pDS35), the *sol5* gene promoter (pII95), or the translation elongation factor 1 alpha gene promoter (pII98). Then the *atr1*, *atr2*, and *atr3* genes were individually cloned to the expression vectors. Details on expression vector construction and cloning procedures are described in Text S1. For heterologous expression of the *atr1* gene, the *sol1*::*atr1*/pDS35 plasmid (5 to 10 μg) was used to transform the “clean host.” Putative transformants were subcultured on potato dextrose agar (PDA; BD Biosciences) containing 100 μg/ml of nourseothricin sulfate (clonNAT; GoldBio). Two transformants, designated Sta04644-T16 and Sta04644-T25, exhibited resistance to the selective agent and were subcultured on PDA overlaid with a nylon membrane for RNA extraction. After 2 weeks of culture, mycelia growing on PDA were scraped off from the nylon membrane with a single-edge razor blade and were subjected to total RNA extraction. Total RNA was extracted from hyphae ground in liquid nitrogen, using TRIzol reagent (Thermo Fisher Scientific) according to the manufacturer's instructions, with the following additional extraction steps: one phenol (pH 4.6)-chloroform-isoamyl alcohol (25:24:1) extraction followed by one chloroform extraction step after the initial TRIzol-chloroform phase separation. RNA pellets were dissolved in 88 μl of nuclease-free water and subjected to genomic DNA digestion by DNase treatment (Qiagen). Then RNA samples were concentrated using the RNA Clean & Concentrator (Zymo Research). We confirmed expression and intron splicing of the *atr1* gene with four introns by RT-PCR analysis. Two primer sets were designed: (i) to amplify a flanking region of the first intron located at the 5′ region of the *atr1* coding sequence and (ii) to amplify a region harboring the second, third, and fourth (the last) introns at the 3′ region of the *atr1* coding sequence. Two hundred nanograms of total RNA was reverse transcribed, and the *atr1* gene and actin 1 (as a positive control) were amplified (39) with a Qiagen OneStep RT-PCR kit. The primers used in RT-PCR analysis and plasmid constructions are listed in Table S2.

### Generation of a heterologous host producing atranorin.

For introduction of tailoring enzymes into a *atr1*-expressing strain (Sta04644-T25), the four resulting plasmids (*sol5*::*atr2*/pII95, *tef1α*::*atr2*/pII98, *sol5*::*atr3*/pII95, and *tef1α*::*atr3*/pII98) were individually transformed into the Sta04644-T25 strain, and putative transformants were subcultured on PDA containing 200 μg/ml of G418 disulfate (Sigma-Aldrich). Transformants resistant to G418 disulfate were selected for metabolite identification. A strain harboring both *sol1*:: *atr1*/pDS35 and *sol5*:: *atr3*/pII95 was used for the generation of an atranorin-producing strain, which exhibited the greatest production of compound 2, the immediate precursor of atrarnoin. Since a neomycin phosphotransferase II gene (*nptII*) cassette that confers resistance to G418 disulfate was already integrated into the strain producing compound 2, we replaced the *nptII* cassette in the *tef1α*::*atr2*/pII98 plasmid with a bleomycin-resistant protein gene (*ble*), the *tef1α*::*atr2*/pII98 plasmid was amplified using a primer pair Inf_BLE_pII99_fwd and Inf_BLE_pII99_rev, and the *ble* gene was amplified from pAC1750 (80). Then the two PCR products were fused using the In-Fusion HD Cloning kit (TaKaRa). The resulting plasmid was transformed into the strain producing compound 2, and putative transformants were subcultured on PDA containing 200 μg/ml of Zeocin (a member of bleomycin family distributed by ThermoFisher Scientific). To select transformants that produce atranorin, the chemical profiles of transformants resistant to Zeocin were compared to that of an *S. alpinum* voucher specimen (Korea National Arboretum accession no. KHL0017342), from which the genome-sequenced LFF had been originally isolated. Detailed methodologies for high-performance liquid chromatography (HPLC), LC-MS/MS, and NMR spectroscopic analyses for culture extracts and purified compounds are fully described in Text S1.

### Data availability.

The data from this whole-genome shotgun project have been deposited in DDBJ/ENA/GenBank under accession no. JAFEKC000000000, JAFEKC000000000, and JAEUBA000000000 (BioProject no. PRJNA693578, PRJNA693575, and PRJNA693574) for *C. borealis*, *Parmelia* cf. *squarrosa*, and *S. alpinum*, respectively. Raw LC-MS/MS data have been deposited in the MassIVE database (http://massive.ucsd.edu) under accession no. MSV000087081.
