# Supplementary material for: Linking a Gene Cluster to Atranorin, a Major Cortical Substance of Lichens, through Genetic Dereplication and Heterologous Expression
Source: mBio. 2021 Jun 22;12(3):e01111-21. doi: 10.1128/mBio.01111-21 (PMC8262933; doi:10.1128/mBio.01111-21)
Supplement: FIG S1 [file mbio.01111-21-sf001.pdf]

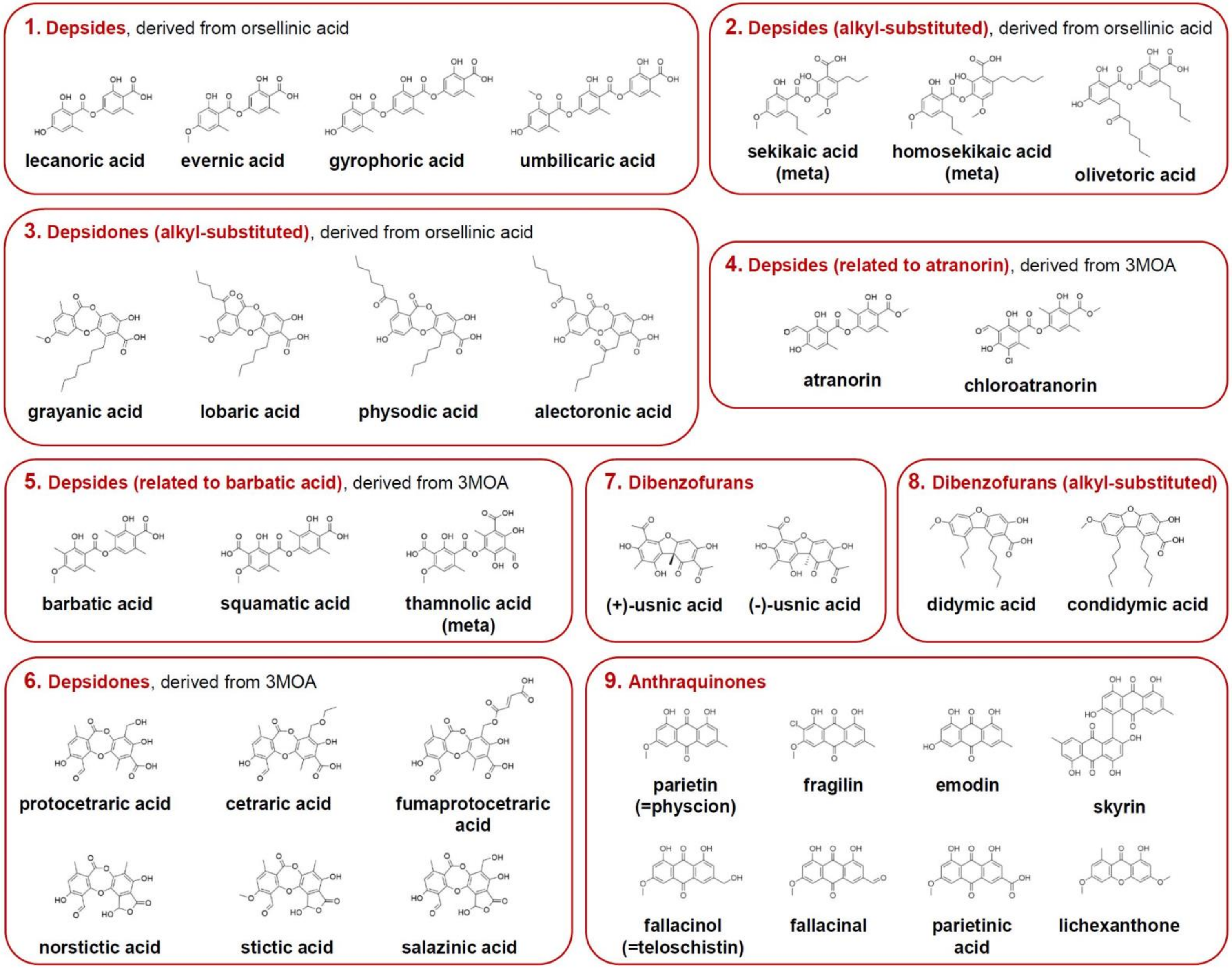


**Fig. S1. Chemical structures of cortical and medullary substances of lichens**. Lichen substances reported in the 30 genome-sequenced species are grouped by nine chemical groups, based on the chemical structures (Also, see Table S1). 3MOA, 3-methylorsellinic acid.
