# Supplementary material for: Linking a Gene Cluster to Atranorin, a Major Cortical Substance of Lichens, through Genetic Dereplication and Heterologous Expression
Source: mBio. 2021 Jun 22;12(3):e01111-21. doi: 10.1128/mBio.01111-21 (PMC8262933; doi:10.1128/mBio.01111-21)
Supplement: TABLE S1 [file mbio.01111-21-st001.docx]

**Table S1.** Lichen substances reported in the 30 genome-sequenced species.

| **Species** | **Lichen substances***^a^* | **Chemical groups***^b^* | **References** |
| --- | --- | --- | --- |
| *Cladonia macilenta* | barbatic acid, didymic acid | 5,8 | Culberson 1970; Stenroos et al. 2002 |
| *Cladonia metacorallifera* | thamnolic acid, **usnic acid**, didymic acid | 5,**7**,8 | Culberson 1970; Stenroos et al. 2002 |
| *Cladonia borealis* | barbatic acid, **usnic acid** | 5,**7** | Stenroos et al. 2002 |
| *Cladonia uncialis* | (squamatic acid), **usnic acid** | (5),**7** | Culberson 1970; Stenroos et al. 2002 |
| *Cladonia rangiferina* | **atranorin,** fumaprotocetraric acid | **4**,6 | Culberson 1970; Stenroos et al. 2002 |
| *Cladonia grayi* | grayanic acid, (fumaprotocetraric acid) | 3,(6) | Culberson 1970; Stenroos et al. 2002 |
| *Cetradonia linearis* | - | - | - |
| *Stereocaulon alpinum* | lobaric acid, **atranorin,** (stictic acid) | 3,**4**,(6) | Culberson 1970 |
| *Letharia lupina* | **atranorin,** norstictic acid | **4**,6 | Altermann et al. 2016 |
| *Letharia columbiana* | **atranorin** | **4** | Altermann et al. 2016 |
| *Pseudevernia furfuracea* | (olivetoric acid), physodic acid, **atranorin/chloroatranorin** | (2),3,**4** | Culberson 1970; Stocker-Wörgötter et al. 2013 |
| *Alectoria sarmentosa* | alectoronic acid, **usnic acid** | 3,**7** | Culberson 1970 |
| *Evernia prunastri* | lecanoric acid/evernic acid, (physodic acid), **atranorin/chloroatranorin,** (salazinic acid), **usnic acid** | 1,(3),**4**,(6),**7** | Culberson 1970; Staple et al. 2010 |
| *Parmelia* sp. KoRLI021559 | **atranorin/chloroatranorin,** salazinic acid | **4**,6 | Culberson 1970 |
| *Usnea florida* | (thamnolic acid), norstictic acid/salazinic acid, **usnic acid** | (5),6,**7** | Culberson 1970; Fiscus 1972 |
| *Usnea hakonensis* | **usnic acid** | **7** | Kono et al. 2020 |

**Table S1.** Continued.

| **Species** | **Lichen substances***^a^* | **Chemical groups***^b^* | **References** |
| --- | --- | --- | --- |
| *Ramalina intermedia* | sekikaic acid, (atranorin), **usnic acid** | 2,(4),**7** | Bowler and Rundel 1974; Oh et al. 2014 |
| *Ramalina peruviana* | sekikaic acid, **usnic acid** | 2,**7** | Bowler and Rundel 1974; Oh et al. 2014 |
| *Xanthoria parietina* | **parietin**/fallacinol/parietinic acid/emodin | **9** | Culberson 1970 |
| *Xanthoria elegans* | **parietin** | **9** | Brunauer et al. 2007 |
| *Gyalolechia flavorubescens* | **parietin**/fallacinal/fragilin/emodin | **9** | Culberson 1970 |
| *Lobaria pulmonaria* | gyrophoric acid, (atranorin), norstictic acid/salazinic acid | 1,(4),6 | Culbersen 1969; Culberson 1970 |
| *Umbilicaria pustulata* | lecanoric acid/gyrophoric acid/umbilicaric acid, skyrin | 1,9 | Culberson 1970; Posner et al. 1991 |
| *Umbilicaria hispanica* | lecanoric acid/gyrophoric acid/umbilicaric acid, skyrin | 1,9 | Culberson 1970; Posner et al. 1991 |
| *Umbilicaria muehlenbergii* | lecanoric acid/gyrophoric acid | 1 | Culberson 1970 |
| *Cyanodermella asteris* | skyrin | 9 | Jahn et al. 2017 |
| *Sclerophora sanguinea* | - | - | - |
| *Arthonia radiata* | none detected | - | Culberson 1970 |
| *Viridothelium virens* | **lichexanthone** | **9** | Aptroot and Lücking 2016 |
| *Endocarpon pusillum* | - | - | - |

*^a^* Cortical substances are labelled in bold, and lichen substances that are found in rare chemotypes are in parenthesis.

*^b^* Lichen substances are grouped by their chemical structure (also see Fig. S1): 1. Depsides, orsellinic acid (OA)-derived; 2. Depsides alkyl-substituted, OA-derived; 3. Depsidones alkyl-substituted, OA-derived; 4. Depsides related to atranorin, 3-methylorsellinic acid (3MOA)-derived; 5. Depsides, related to barbatic acid, 3MOA-derived; 6. Depsidones, 3MOA-derived; 7. Dibenzofurans; 8. Dibenzofurans, alkyl-substituted; 9. Anthraquinones.

**References for Table S1 (alphabetical order)**

Aptroot, A., and Lücking, R. A revisionary synopsis of the Trypetheliaceae (Ascomycota: Trypetheliales). Lichenologist **48**, 763–982 (2016).

Altermann, S. et al. Tidying up the genus *Letharia*: introducing *L. lupina* sp. nov. and a new circumscription for *L. columbiana*. Lichenologist **48**, 423–439 (2016).

Bowler, P.A. and Rundel, P.W. The *Ramalina intermedia* complex in North America. Bryologist **77**, 617–623 (1974).

Brunauer, G. et al. Alterations in secondary metabolism of aposymbiotically grown mycobionts of *Xanthoria elegans* and cultured resynthesis stages. Plant Physiol. Biochem. **45**, 146–151 (2007).

Culberson, C.F. Chemical studies in the genus *Lobaria* and the occurrence of a new tridepside, 4-*O*-methylgyrophoric acid. Bryologist 72 19–27 (1969).

Culberson, C.F. Supplement to "Chemical and Botanical Guide to Lichen Products". Bryologist **73**, 177–377 (1970).

Fiscus, S.A. A survey of the chemistry of the *Usnea florida* group in North America. Bryologist **75**, 299–304 (1972).

Jahn, L. et al. Linking secondary metabolites to biosynthesis genes in the fungal endophyte *Cyanodermella asteris*: The anti-cancer bisanthraquinone skyrin. *J. Biotechnol.* **257**, 233–239. (2017).

Kono, M. et al. In vitro resynthesis of lichenization reveals the genetic background of symbiosis-specific fungal-algal interaction in *Usnea hakonensis*. BMC Genomics **21**, 671 (2020).

Oh, S-.O. et al. A note on the lichen genus *Ramalina* (Ramalinaceae, Ascomycota) in the Hengduan mountains in China. *Mycobiology* **42**, 229-240 (2014).

Posner, B., Feige, G.B., and Leuckert, C. Beiträge zur chemie der flechtengattung *Lasallia* Merat. *Z Naturforsch C* **46**, 19–27 (1991).

Staple, R. et al. Structure and chemical analysis of major specialized metabolites produced by the lichen *Evernia prunastri*. *Chem. Biodivers.* **17**, e1900465 (2020).

Stenroos, S., and Ahti, T.. Phylogeny of the genus *Cladonia* s.lat. (Cladoniaceae, Ascomycetes) inferred from molecular, morphological, and chemical data. *Cladistics* **18**, 237–278 (2002).

Stocker-Wörgötter, E., Cordeiro, L.M.C., and Iacomini, M. Accumulation of potential pharmaceutically relevant lichen metabolites in lichens and cultured lichen symbionts. *Stud. Nat. Prod. Chemistry* **39**, 337-380 (2013).
