# Supplementary material for: Linking a Gene Cluster to Atranorin, a Major Cortical Substance of Lichens, through Genetic Dereplication and Heterologous Expression
Source: mBio. 2021 Jun 22;12(3):e01111-21. doi: 10.1128/mBio.01111-21 (PMC8262933; doi:10.1128/mBio.01111-21)
Supplement: TEXT S1 [file mbio.01111-21-t0001.docx]

**Supplementary Text S1 for**

Linking a gene cluster to atranorin, a major cortical substance of lichens, through genetic dereplication and heterologous expression

Wonyong Kim*, Rundong Liu, Sunmin Woo, Kyo Bin Kang, Hyun Park, Young Hyun Yu, Hyung-Ho Ha, Seung-Yoon Oh, Ji Ho Yang, Hangun Kim, Sung-Hwan Yun, and Jae-Seoun Hur*

*Wonyong Kim; Jae-Seoun Hur

**Email:**  [wykim@scnu.ac.kr;](mailto:xxxxx@xxxx.xxx) jshur1@scnu.ac.kr

| **Contents** | **Page** |
| --- | --- |
| **Supplementary Materials and Methods** | **2** |
| **NMR spectroscopic data summary** | **4** |
| **Notes on *Cladonia* PKS families and genetic dereplication** | **5** |
| **Notes on homology mapping of *Cladonia* PKS families** | **7** |
| **References** | **9** |

**Table of Contents**

Supplementary Materials and Methods

**Genomes sequencing and annotation.** LFF were isolated from *C. borealis* and *S. alpinum* collected from the King George Island, Antarctica (S62˚13'51" W58˚46'32") and *Parmelia* cf. *squarrosa* collected from Mt. Deogyu, South Korea (N35˚51'24" E127˚44'54"). The LFF were cultured for 3–6 months on malt extract agar media (BD Biosciences). Genomic DNAs were extracted using a standard phenol/chloroform extraction method, and were sequenced on an Illumina HiSeq2000 or NovaSeq6000 instrument, generating 150 nucleotide paired-end reads. Illumina adaptors were trimmed, read quality was assessed, and contigs were assembled at Macrogen Inc. (Seoul, South Korea). Details on genome assembly statistics, including previously sequenced genomes, were provided in Data Set S1 in the supplemental material. The genome assemblies retrieved from the NCBI database were annotated using GenSAS (v.6.0) annotation pipeline (1). Default settings were used unless otherwise noted. In brief, low complexity regions and repeats were masked using RepeatModeler (v1.0.11) and RepeatMasker (v4.0.7) (www.repeatmasker.org), setting the DNA source to ‘Fungi’. A masked consensus sequence was generated on which *ab initio* gene prediction was performed using the following tools: (i) Augustus (v3.3.1) (2), selecting *A. nidulans* as a trained organism, (ii) GeneMarkES (v4.33) (3), (iii) Genscan (v1.0) (4), using a parameter setting for Human and other vertebrates, and (iv) GlimmerM (v2.5.1) (5), selecting *Aspergillus* as a trained organism. For homology-based prediction, the NCBI reference transcript and protein databases for Fungi were searched, using (v) BLAST+ (v2.7.1) (6) and (vi) DIAMOND (v0.9.22) (7), respectively. For the consensus gene model prediction using EVidenceModeler (v06-25-2012) (8), the above-mentioned standalone gene predictions were weighted as follows: (i)–five, (ii)-ten, (iii)-one, (iv)-one, (v)-five, and (vi)-five. The genome annotations were assessed with BUSCO (v5b)(9) using the Ascomycota_odb9 data set.

**Expression vector construction for PKS expression.** The pDS23 plasmid carries a nourseothricin acetyltransferase gene (*nat*) cassette as a selection marker and an enhanced green fluorescent protein (*eGFP*) under the control of the constitutive promoter of glyceraldehyde-3-phosphate dehydrogenase gene (*G3PD*) derived from *Aspergillus nidulans* (10). To replace the *G3PD* promoter with multiple cloning sites in pDS23, the plasmid was linearized by AvrII and HindIII restriction enzymes, and a synthetic DNA (57-bp long) harboring several restriction enzyme sites (NheI, AatII, SacI, PmeI, BglII, and SacII) was cloned between the AvrII and HindIII sites of the plasmid. The resulting plasmid was named pDS33. To generate an expression vector that carries a native promoter of *As. rabiei*, the 1,270-bp DNA fragment containing the *sol1* (encoding solanapyrone synthase) gene promoter amplified from gDNA of *As. rabiei* isolate AR628 (11) was cloned to pDS33 precut with BglII and HindIII, yielding the pDS35 plasmid. The coding sequence of *atr1* (Sta04644; see Data Set S2 in the supplemental material) was amplified with a primer pair ‘Inf_Sta046440_fwd’ and ‘Inf_Sta046440_rev’ (7,829 bp), and cloned to the pDS35 linearized by HindIII and BamHI, in replace of eGFP, using the In-Fusion HD Cloning Kit (Takara), which resulted in the plasmid *sol1*::*atr1*/pDS35.

**Expression vector construction for tailoring enzyme expression.** The pII99 plasmid (12) carries a neomycin phosphotransferase II gene (*nptII*) cassette as a selection marker. To generate an expression vectors that carries the promoter of the *sol5* (encoding a Diels-Alderase that catalyzes the last step of solanapyrone biosynthesis) (13), the 579-bp DNA fragment containing the *sol5* promoter amplified from gDNA of *As. rabiei* AR628 strain was cloned to pII99 precut with EcoRI and BglII, yielding the pII95 plasmid. To introduce an additional restriction enzyme site, the revere primer for the *sol5* promoter amplification was designed to have NheI site (see Table S2). The pII95 plasmid was digested with NheI and BglII, and the PCR products of the *atr3* (Sta04642) or *atr2* (Sta04643) amplified from gDNA of *S. alpinum* was fused to the precut plasmid, resulting in the plasmids, *sol5*::*atr3*/pII95 and *sol5*::*atr2*/pII95. Similarly, to generate an expression vectors that carries the constitutive promoter of translation elongation factor 1 alpha gene (*tef1α*) derived from *A. nidulans*, the plasmid pII98 was generated by cloning the 880-bp DNA fragment containing the *tef1α* promoter amplified from the pAC1750 plasmid (14) into pII99, and the coding sequences plus terminator regions of the *atr3* and *atr2* were individually fused into the pII98 precut with NheI and BglII, generating the plasmids, *tef1α*::*atr3*/pII98 and *tef1α*::*atr2*/pII98.

**HPLC analysis and metabolite purification.** For metabolite extraction and profiling, transformants were grown on PDA for 2–3 weeks and the whole agar containing fungal colony was excised from the Petri dish and soaked into 10 ml of ethyl acetate in a 50 ml falcon tube. After sonication for 30 min, an aliquot of 1 ml of solution was transferred to a 1.5 ml microcentrifuge tube, evaporated to dryness, reconstituted with 100 ul of methanol, and subjected to a high performance liquid chromatography (HPLC) analysis. Pieces of the *S. alpinum* voucher specimen (20–30 mg) were soaked into 300 μl of acetone in a 2 ml microcentrifuge tube. After sonication for 30 min, the sample was filtered through 0.4 mm syringe membrane before injection to HPLC. Chemical profiling of transformants was performed using a Prominence Modular LC-20A HPLC instrument (Shimadzu). The samples were analyzed using a YMC-Pack ODS-A (column size, 150 × 4.6 mm; particle size, 5 µm; pore size, 12 nm; at 40 ℃) and using a photodiode array detector at 254 nm (SPD-M20A, range 180–700 nm). The mobile phase was composed of distilled water/trifluoroacetic acid (99.9:0.1, v/v) for pump A and methanol/ trifluoroacetic acid (99.9:0.1, v/v) for pump B. The HPLC analysis was performed using a gradient program at a flow rate of 1.0 ml/min: 0–30 min, 20–100%; 30–40 min, 100%; 40–52 min, 20% of pump B. We used the lichen substance database for HPLC analysis to identify metabolites found in lichen specimens (15). For structural identification, we purified compounds **1**, **2**, and **4** from culture extracts of transformants producing the compounds as major metabolites. Twenty PDA cultures were extracted three times with ethyl acetate. Culture extracts were evaporated to dryness on a Rotavapor (Buchi), reconstituted with methanol, and subjected to preparative HPLC. Separation was achieved on a HPLC at a flow rate of 2.2 ml/min, using the Kromasil C18-column (250 × 10 mm, 5 µm, 10 nm; at 40 ℃), with UV monitoring at 254 nm. The compound **1** was purified using 66% acetonitrile (t*_R_* = 9.86 min; 9.5 mg); **2** was purified using 85% acetonitrile (t*_R_* = 9.77 min; 6.2 mg); **4** was purified using 80% acetonitrile (t*_R_* = 9.60 min; 2.1 mg). Solvents were buffered with 0.1% trifluoroacetic acid. The purified compounds were subjected to NMR spectroscopic analyses.

**LC–MS/MS analysis for identifying products.** Culture extracts were 20-folds diluted with methanol and filtered with a 0.22 μm PTFE filter. The LC–MS analysis was performed on a Waters Acquity I-Class UPLC system coupled to a Waters VION IMS QTOF mass spectrometer (Waters Co.), which was equipped with an electrospray ionization interface. The mobile phase was comprised of 0.1% formic acid in water (pump A) and acetonitrile (pump B). A stepwise gradient method at a constant flow rate of 0.3 ml/min was used to elute the column with the following conditions: 20–100% of pump B (0–9 min), followed by 3 min of washing and 3 min of reconditioning. The injection volume was 2 μl. The column temperature and sample organizer were maintained at 40°C and 20°C, respectively. Tandem MS analyses were performed in data-independent acquisition (MS^E^), negative ion mode with the *m/z* 50–1500 Da range and acquisition times of 0.2 s. The low collision energy for the detection of the precursor ions was set to 6 eV, while the high collision energy for fragmentation was set to 20–40 eV. The ionization conditions were set as follows: the capillary voltage was 2.5 kV, the cone voltage was 40 V, the source temperature was 100°C, the desolvation temperature was 250°C, the cone gas flow was 0 l/h, and the desolvation gas flow was 800 l/h. High-purity nitrogen was used as the nebulizer and auxiliary gas, and argon was used as the collision gas. The [M−H]^−^ ion of leucine enkephalin at *m/z* 554.2615 was used as the lock mass to ensure mass accuracy and reproducibility. MS/MS spectral data matching of major compounds detected in transformants was performed by the feature-based molecular networking workflow (16) in GNPS (17) after the preprocessing using MS-DIAL (18). The analysis results can be accessible via the link below:

<https://gnps.ucsd.edu/ProteoSAFe/status.jsp?task=b015833efff54576b97f76a27affd711>

NMR spectroscopic data summary

**4-*O*-demethylbarbatic acid (1).** ^1^H NMR (400 MHz, CD_3_COCD_3_): *δ*_H_ 11.65 (s, OH), 6.73 (s, 1H), 6.47 (s, 1H), 2.68 (s, 3H), 2.63 (s, 3H), 2.12 (each s, 3H x 2); ^13^C NMR (100 MHz, CD_3_COCD_3_): *δ*_C_ 170.4, 164.0, 163.3, 160.6, 153.0, 140.9, 140.7, 116.7, 116.6, 111.3, 109.6, 109.2, 103.6, 23.8, 23.1, 8.6, 7.3 (see Fig. S4A); ^13^C NMR signals for carboxyl group were not observed or very-low-intensity maybe owing to the long relaxation times of these carbon nuclei, *δ*_C_ of carboxyl group was 174.1 in published data(19). The ^1^H NMR and ^13^C NMR data were comparable to published data(19). HRESIMS *m/z* 345.0972 [M−H]^−^ (calcd. for C_18_H_17_O_7_, 345.0974); the MS/MS spectrum is deposited in the GNPS spectral library and can be accessible via the link below:

<https://gnps.ucsd.edu/ProteoSAFe/gnpslibraryspectrum.jsp?SpectrumID=CCMSLIB00005885123#%7B%7D>.

**Proatranorin I (2).** ^1^H NMR (400 MHz, CDCl_3_): *δ*_H_ 11.90(s, OH), 11.70(s, OH), 6.50(s, 1H), 6.29(s, 1H), 3.96(s, 3H), 2.60(s, 3H), 2.52(s, 3H), 2.12(s, 3H), 2.07(s, 3H); ^13^C NMR (100 MHz, CDCl_3_): *δ*_C_ 172.4, 170.3, 164.2, 162.9, 159.0, 152.6, 140.7, 139.8, 117.0, 116.4, 111.2, 110.1, 109.0, 104.2, 52.3, 24.6, 24.1, 9.4, 7.7 (see Fig. S4B); ^1^H NMR (400 MHz, CDCl_3_) data was comparable to published data(20); ^13^C NMR (100 MHz, CDCl_3_) data was comparable to 4-*O*-demethylbarbatic acid (**1**) data, except that there was one more oxygen-methyl carbon (*δ*_C_ 52.3) in compound **2**. HRESIMS *m/z* 359.1132 [M−H]^−^ (calcd. for C_19_H_19_O_7_, 359.1131); the MS/MS spectrum is deposited in the GNPS spectral library and can be accessible via the link below:

<https://gnps.ucsd.edu/ProteoSAFe/gnpslibraryspectrum.jsp?SpectrumID=CCMSLIB00005885124#%7B%7D>.

**Proatranorin III (4).** ^1^H NMR (400 MHz, CDCl_3_): *δ*_H_ 12.54 (s, OH), 12.45 (s, OH),11.77(s, OH), 10.35(s, CHO), 6.54(s, 1H), 6.40(s, 1H), 2.68(s, 3H), 2.59(s, 3H), 2.08(s, 3H) (see Fig. S4D); ^1^H NMR (400 MHz, CDCl_3_) data was comparable to published data of atranorin (**5**) (20), except that there was missing a methyl group (*δ*_H_ 3.99) at the carboxyl group in (**4**). HRESIMS *m/z* 359.0771 [M−H]^−^ (calcd. for C_18_H_15_O_8_, 359.0767); the MS/MS spectrum is deposited in the GNPS spectral library and can be accessible via the link below:

<https://gnps.ucsd.edu/ProteoSAFe/gnpslibraryspectrum.jsp?SpectrumID=CCMSLIB00005885126#%7B%7D>.

Notes on *Cladonia* PKS families and genetic dereplication

**PKS families in the genus *Cladonia*.** Forty-five gene cluster families (GCFs) including at least one iterative type I PKS gene were identified, using the Big-SCAPE program. Biosynthetic gene clusters (BGCs) of PKS1, PKS2, PKS4, PKS5, PKS15 and PKS16 families were conserved in the six *Cladonia* species plus *S. alpinum*, and BGCs of PKS3 and PKS10 families were conserved in the six *Cladonia* species. Previously, Timsina *et al.* (21) identified thirteen PKS families conserved in species in the *Cladonia chlorophaea* species complex (PKS1, PKS2, PKS3, PKS5, PKS7, PKS10, PKS11, PKS12, PKS13, PKS14, PKS15, PKS16 and MSAS). We also identified these PKS families in our gene cluster network analysis (Fig. 2A). However, we failed to detect PKS12 homologs in the genomes of the six *Cladonia* species, even in the *C. grayi* genome from which the PKS12 family was originally identified. Three PKSs are manually detected and linked to respective PKS families, as the gene annotations were incomplete and fragmented so that the antiSMASH program failed to detect BGCs harboring the three PKSs: Cma06514 (PKS3), Cgr07347 (PKS10), and Cuc08442 (PKS19) (see Data Set S2).

**Genetic dereplication of *Cladonia* BGCs.** To link unknown biosynthetic gene clusters in the six *Cladonia* spp. and *S. alpinum* to known compounds in other fungi, GCFs predicted by the Big-SCAPE program were coupled to previously characterized BGCs deposited in the Minimal Information about a Biosynthetic Gene cluster (MIBiG) database (v.1.4) (22). As a result, eight GCFs and seven singleton BGCs were linked to known BGCs found in non-lichenized fungi (Fig. 2A). These conserved biosynthetic gene clusters (BGCs) in phylogenetically distant species suggested shared ancestries of the biosynthetic pathways between lichens and non-lichenized fungi. Nevertheless, these conserved BGCs exhibited a high degree of gene content variation, suggesting divergent pathways that may have led to chemical diversity. There were three lichen BGCs listed in the MIBiG database: BGCs for PKS8 in *C. uncialis* (23), PKS14 in *C. uncialis* (24), and PKS16 in *C. grayi* (25)*,* which were indicated by dashed red circles in Fig. 2A.

**PKS8.** The PKS8, also known as methylphloroacetophenone synthase (MPAS) in *C. uncialis,* is known to be involved in biosynthesis of usnic acid, based on phylogenetic analysis and gene expression study (23, 26). A GCF of the PKS8/MPAS family is shared by *C. borealis*, *C. metacorallifera, C. rangiferina,* and *C. uncialis*. However, among these four *Cladonia* species, usnic acid has not been detected in *C. rangiferina* (27, 28) (see Fig. S5B in the supplemental material)*.*

**PKS14.** The putative 6-hydroxymellein BGC found in *C. uncialis* (BGC0001489) formed a GCF with PKS14 BGCs in *C. borealis* and *C. grayi*. The roles of the gene members of the 6-hydroxymellein BGC were tentatively assigned based on the terrein biosynthetic pathway (24), in which *terA* and *terB* act in concert to biosynthesize 6-hydroxymellein in *A. terreus* (29). In our network analysis, the terrein BGC (MIBiG accession: BGC0000161) was not linked to the 6-hydroxymellein BGCs in the three *Cladonia* species, likely due to gene content variation observed between them; only three genes (*terA*, *terB* and *terD*) among the eleven gene members of the terrein BGC in *A. terreus* were conserved in the 6-hydroxymellein BGCs in the three *Cladonia* species.

**PKS13 and PKS15.** BGCs of the PKS15 family conserved in the six *Cladonia* spp. and *S. alpinum* were linked to many previously characterized BGCs including a 1,3,6,8-tetrahydroxynaphthalene (T4HN) synthase responsible for melanin production in diverse fungi (see Data Set S2). For brevity, Figure 2A showed only one BGC containing a T4HN synthase (BGC0001257) connected to the BGCs of the PKS15 family. We identified that PKS13 BGCs were also connected to known BGCs containing a T4HN synthase. Unlike the PKS15 family conserved in the six *Cladonia* spp. and *S. alpinum*, the PKS13 family was not found in *C. uncialis* and *S. alpinum*, showing 51–54% protein sequence identity with ElcPKS1 responsible for the melanin production in *Exophiala lecanii-corni* (30, 31) (Fig. 5). The ElcPKS1 and PKS13 family formed a highly supported clade (bootstrap value of 99%) distinct from the PKS15 family and other conventional T4HNs, indicating that some *Cladonia* species possess two copies of melanin PKSs of different origin.

**PKS16.** The putative grayanic BGC found in *C. grayi* (BGC0001266) is conserved in the six *Cladonia* spp. and *S. alpinum*. The PKS16 BGC was proposed to produce orsellinic acid-derived grayanic acid (a depsidone) that is found in *C. grayi* (25).

**PKS17.** A GCF of the PKS17 family, shared by *C. borealis*, *C. macilenta* and *C. metacorallifera*, was linked to a BGC (BGC0001583) for the biosynthesis of emodin, an anthraquinone produced by many different fungi. In addition to the four core enzymes (an NR-PKS, metallo-*β*-lactamase, decarboxylase and anthrone oxygenase) that are necessary and sufficient for emodin biosynthesis, a few more tailoring enzymes including a cytochrome P450 monooxygenase were found in the homologous *Cladonia* BGCs. The P450 monooxygenase showed 39% sequence identity with the ClaM that mediates the coupling of two emodin derivatives in *Cladosporium fulvum* (32), suggesting that the P450 enzyme may be involved in biosynthesis of skyrin (33). Intriguingly, another BGC harboring the four core genes for emodin biosynthesis was found in the genome of *C. macilenta*, although in different arrangement with the PKS17 BGCs. This second BGC in *C. macilenta* for putative emodin biosynthesis formed a GCF with the PKS18 BGC in *C. uncialis*, which was homologous to the BGC of pestheic acid, an emodin derivative, found in *Pestalotiopsis fici* (34).

**PKS34.** A GCF of the PKS34 family shared by *C. macilenta* and *C. metacorallifera* was linked to a BGC (BGC0001242) for the biosynthesis of fusarubin, the red mycelial pigment produced by *Fusarium fujikuroi* (35). However, the BGC contents were highly variable; only putative homologs of *fsr1* (encoding an NR-PKS), *fsr4* (encoding an alcohol dehydrogenase) and *fsr5* (encoding a short-chain dehydrogenase) in the fusarubin BGC can be found in the PKS34 BGCs.

**PKS37.** The patulin BGC (BGC0000120) in *Penicillium expansum* was connected to a GCF of the PKS37 family shared by *C. borealis*, *C. metacorallifera* and *C. uncialis*. Among the 15 gene members of the patulin BGC, ten genes homologous to *patF*–*patO* can be found in the three *Cladonia* spp. (except for *patF* in *C. borealis*), which are necessary for the biosynthesis of neopatulin, the immediate precursor of patulin (36). Homologs of *patD* and *patE* responsible for the final conversion of neopatulin to patulin were absent in the *Cladonia* genomes. Instead, other tailoring enzymes found were an epimerase in *C. metacorallifera* and *C. uncialis*, a TauD-like dioxygenase in *C. borealis* and *C. uncialis*, and two short chain dehydrogenases in *C. uncialis*.

**Singleton BGCs connected to the MIBiG database.** Several previously characterized BGCs were connected to BGCs found in a single species (singleton BGCs). These singleton BGCs tend to exhibit little gene content variation and high sequence similarity to corresponding BGCs found in non-lichenized fungi, suggesting recent introductions of these BGCs into lichens. These likely horizontally transferred BGCs from non-lichenized fungi were as follows: the sorbicillin BGC (BGC0001404) linked to a BGC in *C. macilenta*, the terreic acid BGC (BGC0000160) linked to a BGC in *C. grayi*, the hypothemycin BGC (BGC0000076) linked to a BGC in *C. rangiferina*, the curvupallides BGC (BGC0001563) linked to a BGC in *C. uncialis*, and the depudecin BGC (BGC0000046) linked to a BGC in *S. alpinum*. The monascorubrin BGC (BGC0000099) and betaenones BGC (BGC0001264) were also connected to BGCs in *C. uncialis* in our network analysis, as well as in an earlier study on homology mapping of *C. uncialis* PKSs (37).

Notes on homology mapping of *Cladonia* PKS families

**Homology mapping of conserved lichen PKS families.** Although gene cluster network analysis ascribed many lichen BGCs to known compounds with high likelihood, there may be more homologous BGCs that were not connected to previously characterized BGCs in non-lichenized fungi, due to high gene content variations. Therefore, we performed BLAST searches of 45 PKS families against the NCBI non-redundant protein sequences database to identify *Cladonia* PKS families homologous to PKS genes with known compounds.

**PKS4.** The PKS4 family encodes an R-PKS enzyme conserved in the six *Cladonia* spp. and *S. alpinum*. The PKS4 family can be found in lichenized and non-lichenized fungi, and appears to have undergone co-evolution with the physically-associated type III PKS (38).

**PKS5.** The PKS5 family was conserved in the six *Cladonia* spp. and *S. alpinum*, encoding a PKS–NRPS hybrid enzyme that showed a high sequence similarity to the xenolozoyenone PKS in *Glarea lozoyensis* (39) (56% at protein sequence level). Homologs of the PKS5 family can be found in diverse fungi (39). Interestingly, two tandem genes encoding R-PKS and NRPS enzymes for the xenolozoyenone biosynthesis in *G. lozoyensis* are co-transcribed into one dicistronic mRNA under the control of the same promoter. Thus, it needs to be examined whether the PKS5 family in lichens also encodes two separate polypeptides for R-PKS and NRPS enzymes or a single peptide for a PKS–NRPS hybrid enzyme.

**PKS9.** The PKS9 family was shared by *C. borealis*, *C. macilenta*, *C. metacorallifera* and *C. uncialis*, which is similar to an NR-PKS (MpaC) responsible for the biosynthesis of mycophenolic acid derived from 5-methylorsellinic acid in *Penicillium brevicompactum* (44% protein sequence identity), as described in a previous homology mapping study (37). Also, the PKS9 family showed 43–45% protein sequence identity to NR-PKSs (AdrD, AndM, AusA, NvfA, PrhL and Trt4; see Data Set S2) involved in the biosynthesis of meroterpenoids derived from 3,5-dimethylorsellinic acid in *Aspergillus* and *Penicillium* species (40–45). However, the PKS9 BGCs in the *Cladonia* genomes lacked biosynthetic genes related to terpene biosynthesis, such as prenyltransferase and terpene cyclase.

**PKS18.** The PKS18 family shared by in *C. macilenta* and *C. uncialis* showed 64–68% protein sequence identity to an NR-PKS (PtaA) involved in the biosynthesis of pestheic acid, an emodin derivative, in *Pestalotiopsis fici* (34), as described in a previous homology mapping study (37).

**PKS21.** A purple pigment biruloquinone was first discovered in a foliose lichen *Parmelia birulae* (46). Although biruloquinone has not been found in *Cladonia* species in nature, we previously identified a chemical variant of the genome-sequenced mycobiont isolated from *C. macilenta*, producing biruloquinone in axenic culture (47). We tentatively assigned the PKS21 family to the biosynthesis of biruloquinone by the whole-transcriptome comparison between the biruloquinone producer and non-producer (48).

**PKS22.** A red pigment cristazarin, a naphthazarin derivative, was originally identified from a mycobiont culture of *C. cristatella* (49). We established an optimum culture condition for cristazarin production in a mycobiont isolated from the genome-sequenced *C. metacorallifera*, and ascribed the PKS22 family to the biosynthesis of cristazarin, based on the observation that the PKS23 was highly upregulated in cristazarin-inducing conditions in *C. metacorallifera* (W. Kim and J-.S. Hur, *unpublished*).

**PKS24.** The PKS24 family was shared by *C. borealis*, *C. macilenta*, *C. metacorallifera* and *S. alpinum.* The PKS24 BGC in *C. metacorallifera* was slightly different from the ones in the other three species, with a fragmented PKS24 gene. The PKS24 family formed a monophyletic clade maximally supported by 100% bootstrap with FgPKS14 involved in orsellinic acid biosynthesis in *Fusarium graminearum* (50), showing 46–48% protein sequence identity to FgPKS14 (Fig. 5).

**PKS30.** The PKS30 family showed 52–53% protein sequence identity to an NR-PKS (wA) responsible for the biosynthesis of the conidial yellow pigment (*aka.* YWA1), a naphthopyrone produced by *A. nidulans* (see Data Set S2). The PKS30 BGCs conserved in *C. borealis*, *C. macilenta*, *C. metacorallifera* and *C. uncialis* are likely involved in the biosynthesis of the red apothecial pigment, rhodocladonic acid, albeit the pigment has not been detected in *C. uncialis*. There were only two GCFs (the PKS21 and PKS30 families) shared by the three red apothecial pigment producers, *C. borealis*, *C. macilenta* and *C. metacorallifera*. The PKS21 and PKS30 families belong to the NR-PKS Groups III and IV, respectively, which include NR-PKSs involved in the biosynthesis of naphthopyrone-derived compounds (Fig. 5). Since the PKS21 family was tentatively assigned to the biosynthesis of biruloquinone (a phenanthrenequinone), the PKS30 family is the last remaining naphthopyrone PKSs shared by the three red apothecial pigment producers. This putative assignment was strengthened by the presence of enzymes necessary for the biosynthesis of rhodocladonic acid, such as *O*-methyltransferase and two monooxygenases in the PKS30 BGCs. A proposed biosynthetic pathway for rhodocladonic acid from a naphthopyrone precursor was shown below.


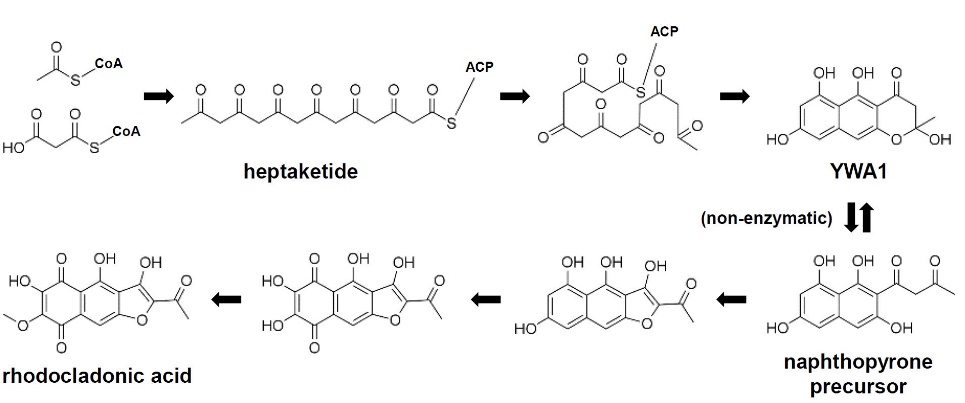


**PkeA.** BGCs shared by *C. borealis* and *C. metacorallifera* included an NR-PKS showing 63–65% protein sequence identity to PKeA (also known as DbaI) responsible for the biosynthesis of felinones in *A. nidulans* (see Data Set S2). We refrained from assigning a PKS family number for the NR-PKSs homologous to PkeA/DbaI, as these PKS genes appear to have arisen through horizontal transfer, and may be specific to a certain lineage of *Cladonia*, as with the other *Cladonia* NR-PKSs that belong to the Group VII (*e.g.* sorbicillin BGC in *C. macilenta*).

.References

1. Humann JL, Lee T, Ficklin S, Main D. 2019. Structural and functional annotation of eukaryotic genomes with GenSAS. Methods Mol Biol 1962:29–51.

2. Stanke M, Waack S. 2003. Gene prediction with a hidden Markov model and a new intron submodel. Bioinformatics 19:ii215–ii225.

3. Ter-Hovhannisyan V, Lomsadze A, Chernoff YO, Borodovsky M. 2008. Gene prediction in novel fungal genomes using an *ab initio* algorithm with unsupervised training. Genome Res 18:1979–1990.

4. Richmond T. 2000. Identification of complete gene structures in genomic DNA. Genome Biol 1:reports222.

5. Delcher AL, Harmon D, Kasif S, White O, Salzberg SL. 1999. Improved microbial gene identification with GLIMMER. Nucleic Acids Res 27:4636–4641.

6. Camacho C, Coulouris G, Avagyan V, Ma N, Papadopoulos J, Bealer K, Madden TL. 2009. BLAST+: architecture and applications. BMC Bioinformatics 10:421.

7. Buchfink B, Xie C, Huson DH. 2015. Fast and sensitive protein alignment using DIAMOND. 1. Nat Methods 12:59–60.

8. Haas BJ, Salzberg SL, Zhu W, Pertea M, Allen JE, Orvis J, White O, Buell CR, Wortman JR. 2008. Automated eukaryotic gene structure annotation using EVidenceModeler and the Program to Assemble Spliced Alignments. Genome Biol 9:R7.

9. Simão FA, Waterhouse RM, Ioannidis P, Kriventseva EV, Zdobnov EM. 2015. BUSCO: assessing genome assembly and annotation completeness with single-copy orthologs. Bioinformatics 31:3210–3212.

10. Teichert I, Wolff G, Kück U, Nowrousian M. 2012. Combining laser microdissection and RNA-seq to chart the transcriptional landscape of fungal development. BMC Genomics 13:511.

11. Kim W, Park J-J, Gang DR, Peever TL, Chen W. 2015. A novel type pathway-specific regulator and dynamic genome environments of a solanapyrone biosynthesis gene cluster in the fungus *Ascochyta rabiei*. Eukaryot Cell 14:1102–1113.

12. Namiki F, Matsunaga M, Okuda M, Inoue I, Nishi K, Fujita Y, Tsuge T. 2001. Mutation of an arginine biosynthesis gene causes reduced pathogenicity in *Fusarium oxysporum* f. sp. *melonis*. Mol Plant Microbe Interact 14:580–584.

13. Kim W, Park C-M, Park J-J, Akamatsu HO, Peever TL, Xian M, Gang DR, Vandemark G, Chen W. 2014. Functional analyses of the Diels-Alderase gene *sol5* of *Ascochyta rabiei* and *Alternaria solani* indicate that the solanapyrone phytotoxins are not required for pathogenicity. Mol Plant Microbe Interact 28:482–496.

14. Vanegas KG, Jarczynska ZD, Strucko T, Mortensen UH. 2019. Cpf1 enables fast and efficient genome editing in *Aspergilli*. Fungal Biol Biotechnol 6:6.

15. Yoshimura I, Kinoshita Y, Yamamoto Y, Huneck S, Yamada Y. 1994. Analysis of secondary metabolites from lichen by high performance liquid chromatography with a photodiode array detector. Phytochem Anal 5:197–205.

16. Nothias L-F, et al. 2020. Feature-based molecular networking in the GNPS analysis environment. 9. Nat Methods 17:905–908.

17. Wang M, et al. 2016. Sharing and community curation of mass spectrometry data with Global Natural Products Social Molecular Networking. 8. Nat Biotechnol 34:828–837.

18. Tsugawa H, Cajka T, Kind T, Ma Y, Higgins B, Ikeda K, Kanazawa M, VanderGheynst J, Fiehn O, Arita M. 2015. MS-DIAL: data-independent MS/MS deconvolution for comprehensive metabolome analysis. 6. Nat Methods 12:523–526.

19. Parrot D, Jan S, Baert N, Guyot S, Tomasi S. 2013. Comparative metabolite profiling and chemical study of *Ramalina siliquosa* complex using LC–ESI-MS/MS approach. Phytochemistry 89:114–124.

20. Zhang S, Meng L, Ma L, Wang J, Zhao M. 2012. Chemical constituents of *Cladoniae stellardis* (Opiz.). Chinese Traditional Patent Medicine 34:297–300.

21. Timsina BA, Hausner G, Piercey-Normore MD. 2014. Evolution of ketosynthase domains of polyketide synthase genes in the *Cladonia chlorophaea* species complex (Cladoniaceae). Fungal Biol 118:896–909.

22. Medema MH, et al. 2015. Minimum Information about a Biosynthetic Gene cluster. Nat Chem Biol 11:625–631.

23. Abdel-Hameed M, Bertrand RL, Piercey-Normore MD, Sorensen JL. 2016. Putative identification of the usnic acid biosynthetic gene cluster by de novo whole-genome sequencing of a lichen-forming fungus. Fungal Biol 120:306–316.

24. Abdel-Hameed M, Bertrand RL, Piercey-Normore MD, Sorensen JL. 2016. Identification of 6-hydroxymellein synthase and accessory genes in the lichen *Cladonia uncialis*. J Nat Prod 79:1645–1650.

25. Armaleo D, Sun X, Culberson C. 2011. Insights from the first putative biosynthetic gene cluster for a lichen depside and depsidone. Mycologia 103:741–754.

26. Wang Y, Geng C, Yuan X, Hua M, Tian F, Li C. 2018. Identification of a putative polyketide synthase gene involved in usnic acid biosynthesis in the lichen *Nephromopsis pallescens*. PLoS ONE 13:e0199110.

27. Stenroos S, Ahti T. 2002. Phylogeny of the genus *Cladonia* s.lat. (Cladoniaceae, Ascomycetes) inferred from molecular, morphological, and chemical data 18:237–278.

28. Elshobary ME, Becker MG, Kalichuk JL, Chan AC, Belmonte MF, Piercey-Normore MD. 2018. Tissue-specific localization of polyketide synthase and other associated genes in the lichen, *Cladonia rangiferina*, using laser microdissection. Phytochemistry 156:142–150.

29. Zaehle C, Gressler M, Shelest E, Geib E, Hertweck C, Brock M. 2014. Terrein biosynthesis in *Aspergillus terreus* and its impact on phytotoxicity. Chem Biol 21:719–731.

30. Wheeler MH, Abramczyk D, Puckhaber LS, Naruse M, Ebizuka Y, Fujii I, Szaniszlo PJ. 2008. New biosynthetic step in the melanin pathway of *Wangiella* (*Exophiala*) *dermatitidis*: Evidence for 2-acetyl-1,3,6,8-tetrahydroxynaphthalene as a novel precursor. Eukaryot Cell 7:1699–1711.

31. Cheng Q, Kinney KA, Whitman CP, Szaniszlo PJ. 2004. Characterization of two polyketide synthase genes in *Exophiala lecanii-corni*, a melanized fungus with bioremediation potential. Bioorg Chem 32:92–108.

32. Griffiths S, Mesarich CH, Saccomanno B, Vaisberg A, Wit PJGMD, Cox R, Collemare J. 2016. Elucidation of cladofulvin biosynthesis reveals a cytochrome P450 monooxygenase required for anthraquinone dimerization. Proc Natl Acad Sci U S A 113:6851–6856.

33. Jahn L, Schafhauser T, Wibberg D, Rückert C, Winkler A, Kulik A, Weber T, Flor L, van Pée K-H, Kalinowski J, Ludwig-Müller J, Wohlleben W. 2017. Linking secondary metabolites to biosynthesis genes in the fungal endophyte *Cyanodermella asteris* : The anti-cancer bisanthraquinone skyrin. J Biotechnol 257:233–239.

34. Xu X, Liu L, Zhang F, Wang W, Li J, Guo L, Che Y, Liu G. 2014. Identification of the first diphenyl ether gene cluster for pestheic acid biosynthesis in plant endophyte *Pestalotiopsis fici*. ChemBioChem 15:284–292.

35. Studt L, Wiemann P, Kleigrewe K, Humpf H-U, Tudzynski B. 2012. Biosynthesis of fusarubins accounts for pigmentation of *Fusarium fujikuroi* perithecia. Appl Environ Microbiol 78:4468–4480.

36. Tannous J, El Khoury R, Snini SP, Lippi Y, El Khoury A, Atoui A, Lteif R, Oswald IP, Puel O. 2014. Sequencing, physical organization and kinetic expression of the patulin biosynthetic gene cluster from *Penicillium expansum*. Int J Food Microbiol 189:51–60.

37. Bertrand RL, Abdel-Hameed M, Sorensen JL. 2018. Lichen biosynthetic gene clusters part II: Homology mapping suggests a functional diversity. J Nat Prod 81:732–748.

38. Navarro-Muñoz JC, Collemare J. 2020. Evolutionary histories of type III polyketide synthases in fungi. Front Microbiol 10:3018.

39. Yue Q, Chen L, Li Y, Bills GF, Zhang X, Xiang M, Li S, Che Y, Wang C, Niu X, An Z, Liu X. 2015. Functional operons in secondary metabolic gene clusters in *Glarea lozoyensis* (Fungi, Ascomycota, Leotiomycetes). mBio 6:e00703-15.

40. Matsuda Y, Awakawa T, Abe I. 2013. Reconstituted biosynthesis of fungal meroterpenoid andrastin A. Tetrahedron 69:8199–8204.

41. Lo H-C, Entwistle R, Guo C-J, Ahuja M, Szewczyk E, Hung J-H, Chiang Y-M, Oakley BR, Wang CCC. 2012. Two separate gene clusters encode the biosynthetic pathway for the meroterpenoids austinol and dehydroaustinol in *Aspergillus nidulans*. J Am Chem Soc 134:4709–4720.

42. Matsuda Y, Awakawa T, Itoh T, Wakimoto T, Kushiro T, Fujii I, Ebizuka Y, Abe I. 2012. Terretonin biosynthesis requires methylation as essential step for cyclization. ChemBioChem 13:1738–1741.

43. Matsuda Y, Wakimoto T, Mori T, Awakawa T, Abe I. 2014. Complete biosynthetic pathway of anditomin: Nature’s sophisticated synthetic route to a complex fungal meroterpenoid. J Am Chem Soc 136:15326–15336.

44. Matsuda Y, Iwabuchi T, Fujimoto T, Awakawa T, Nakashima Y, Mori T, Zhang H, Hayashi F, Abe I. 2016. Discovery of key dioxygenases that diverged the paraherquonin and acetoxydehydroaustin pathways in *Penicillium brasilianum*. J Am Chem Soc 138:12671–12677.

45. Matsuda Y, Bai T, Phippen CBW, Nødvig CS, Kjærbølling I, Vesth TC, Andersen MR, Mortensen UH, Gotfredsen CH, Abe I, Larsen TO. 2018. Novofumigatonin biosynthesis involves a non-heme iron-dependent endoperoxide isomerase for orthoester formation. Nat Commun 9:2587.

46. Arnone A, Nasini G, de Pava OV. 1991. A reinvestigation of the structure of biruloquinone, a 9,10-phenanthrenequinone isolated from *Mycosphaerella rubella*. Phytochemistry 30:2729–2731.

47. Jeong M-H, Kim JA, Yu NH, Jung JS, Hong SG, Cheong YH, Hur J-S. 2015. Isolation and characterization of a non-reducing polyketide synthase gene in *Cladonia macilenta*. Mycoscience 56:49–57.

48. Kim W, Jeong M-H, Yun S-H, Hur J-S. 2021. Transcriptome analysis identifies a gene cluster for the biosynthesis of biruloquinone, a rare phenanthraquinone, in a lichen-forming fungus *Cladonia macilenta*. J Fungi 7:398.

49. Yamamoto Y, Matsubara H, Kinoshita Y, Kinoshita K, Koyama K, Takahashi K, Ahmadjiam V, Kurokawa T, Yoshimura I. 1996. Naphthazarin derivatives from cultures of the lichen *Cladonia cristatella*. Phytochemistry 43:1239–1242.

50. Jørgensen SH, Frandsen RJN, Nielsen KF, Lysøe E, Sondergaard TE, Wimmer R, Giese H, Sørensen JL. 2014. *Fusarium graminearum* PKS14 is involved in orsellinic acid and orcinol synthesis. Fungal Genet Biol 70:24–31.
