# Supplementary material for: Linking a Gene Cluster to Atranorin, a Major Cortical Substance of Lichens, through Genetic Dereplication and Heterologous Expression
Source: mBio. 2021 Jun 22;12(3):e01111-21. doi: 10.1128/mBio.01111-21 (PMC8262933; doi:10.1128/mBio.01111-21)
Supplement: FIG S2 [file mbio.01111-21-sf002.pdf]

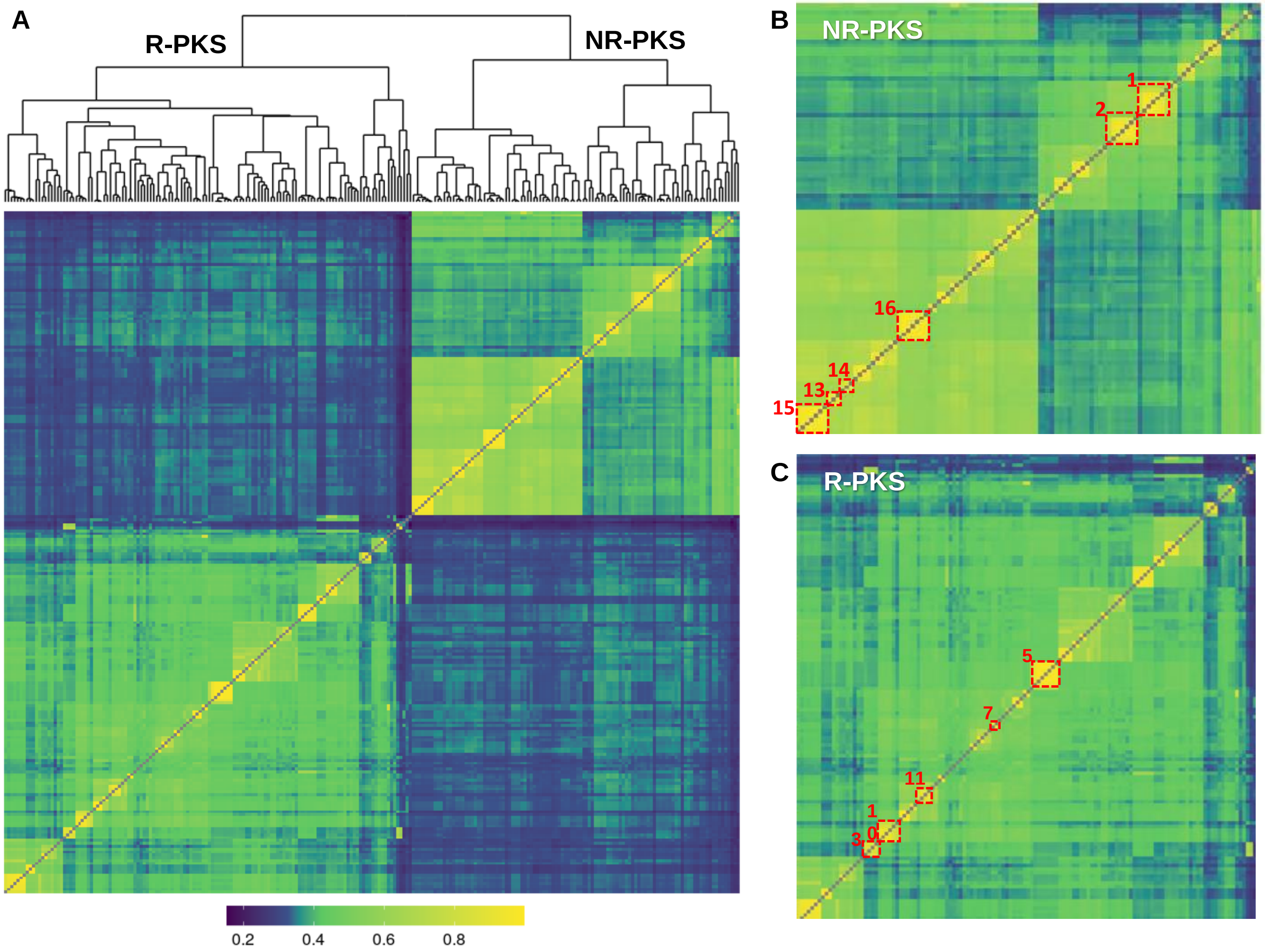

Fig. S2. Overview of the similarity of PKS families. (A) All versus all comparison of ketosynthase domain of 238 PKSs in the six *Cladonia* spp. and *S. alpinum* identifies clusters of PKSs (yellow squares). Enlarged views of clusters of NR-PKS (B) and R-PKS (*C*). Red boxes and associated numbers indicate PKS family identified by Timsina *et al*. 2014.
