# Supplementary material for: Linking a Gene Cluster to Atranorin, a Major Cortical Substance of Lichens, through Genetic Dereplication and Heterologous Expression
Source: mBio. 2021 Jun 22;12(3):e01111-21. doi: 10.1128/mBio.01111-21 (PMC8262933; doi:10.1128/mBio.01111-21)
Supplement: FIG S3 [file mbio.01111-21-sf003.pdf]

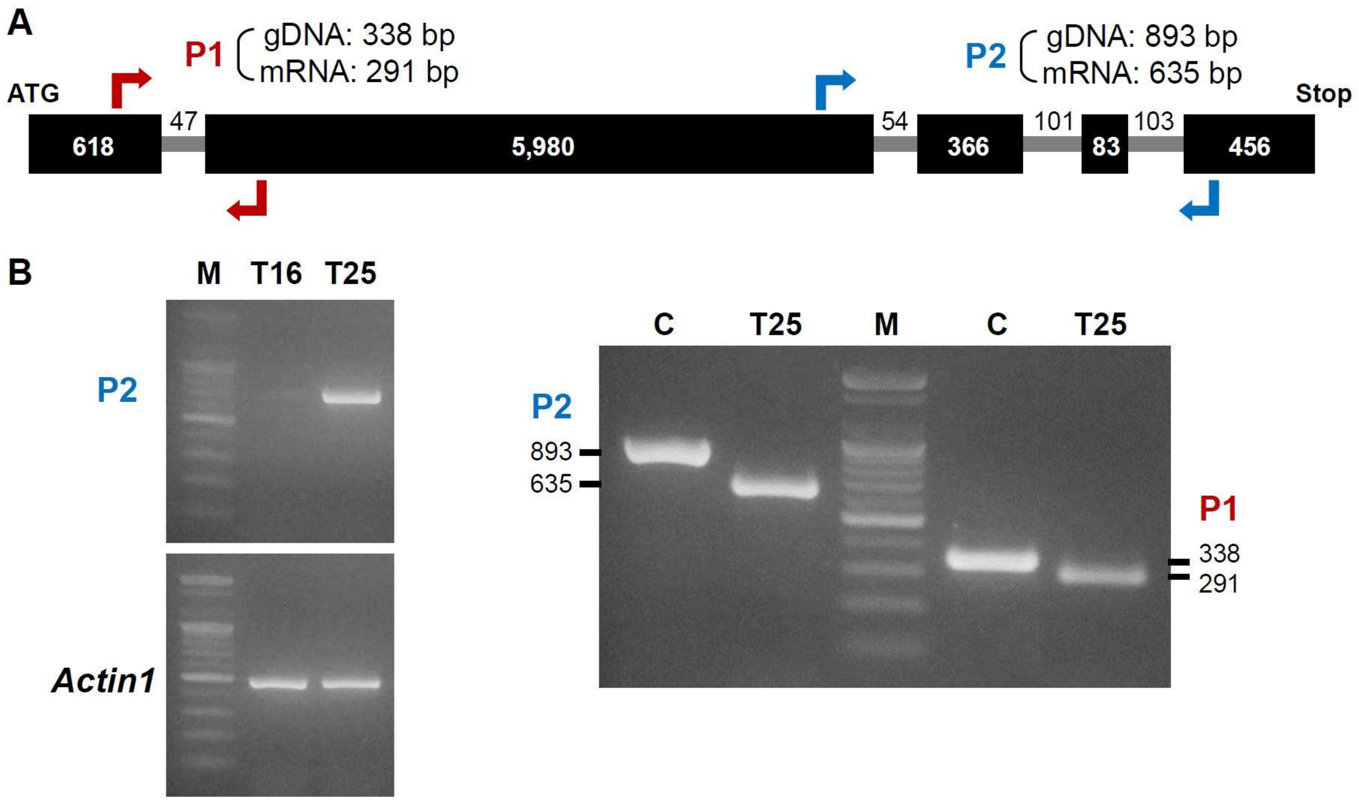

Fig. S3. Verification of heterologous expression of the PKS23. (**A**) A schematic diagram of the PKS23 (*atr1*) gene structure in *Stereocaulon alpinum*. Black boxes are exons, and grey lines are introns. The numbers indicate the size of exons and introns in base pairs (bp). P1 and P2 are primer pairs used in RT-PCR analyses. These primer pairs are designed to include one or more intron regions so that mRNA expression can be distinguished from genomic DNA (gDNA) amplification. (**B**) RT-PCR analyses of two putative transformants (T16 and T25) carrying the plasmid *sol1*::*atr1*/pDS35. No band corresponding to gDNA amplicon for Actin1 reference gene (633 bp; *cf*. 482 bp for mRNA; see ref. (11)) is observed, indicating that there was no genomic DNA contamination (lower left panel). The transformant T25 shows stronger expression of the introduced *atr1* than the transformant T16 (upper left panel). Splicing of the four introns in the *atr1* is confirmed by comparison of band sizes between the plasmid *sol1*::*atr1*/pDS35 (C) and RNA sample (T25), using P1 and P2 primer pairs. The expected band sizes for mRNA expression and plasmid DNA amplification are indicated by numbers to left and right sides of the gel image in bp. M, 100 bp DNA ladder.
