# Supplementary material for: Linking a Gene Cluster to Atranorin, a Major Cortical Substance of Lichens, through Genetic Dereplication and Heterologous Expression
Source: mBio. 2021 Jun 22;12(3):e01111-21. doi: 10.1128/mBio.01111-21 (PMC8262933; doi:10.1128/mBio.01111-21)
Supplement: FIG S4 [file mbio.01111-21-sf004.pdf]

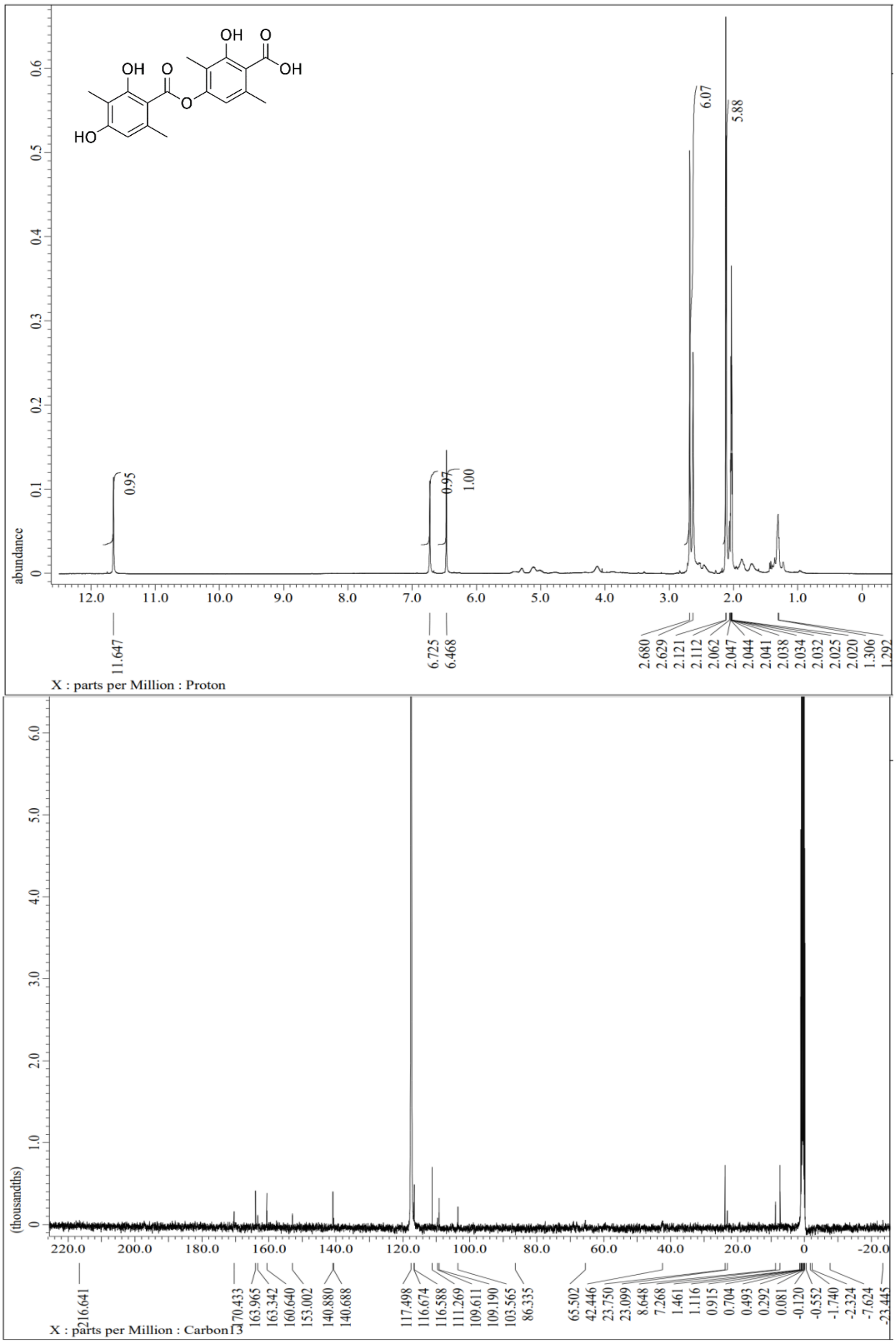

Fig. S4A. NMR analysis for 4-*O*-demethylbarbatic acid. ^{1}H-NMR (400 MHz; upper panel) and ^{13}C-NMR (100 MHz; lower panel) spectra of **1** in CD_3COCD_3.

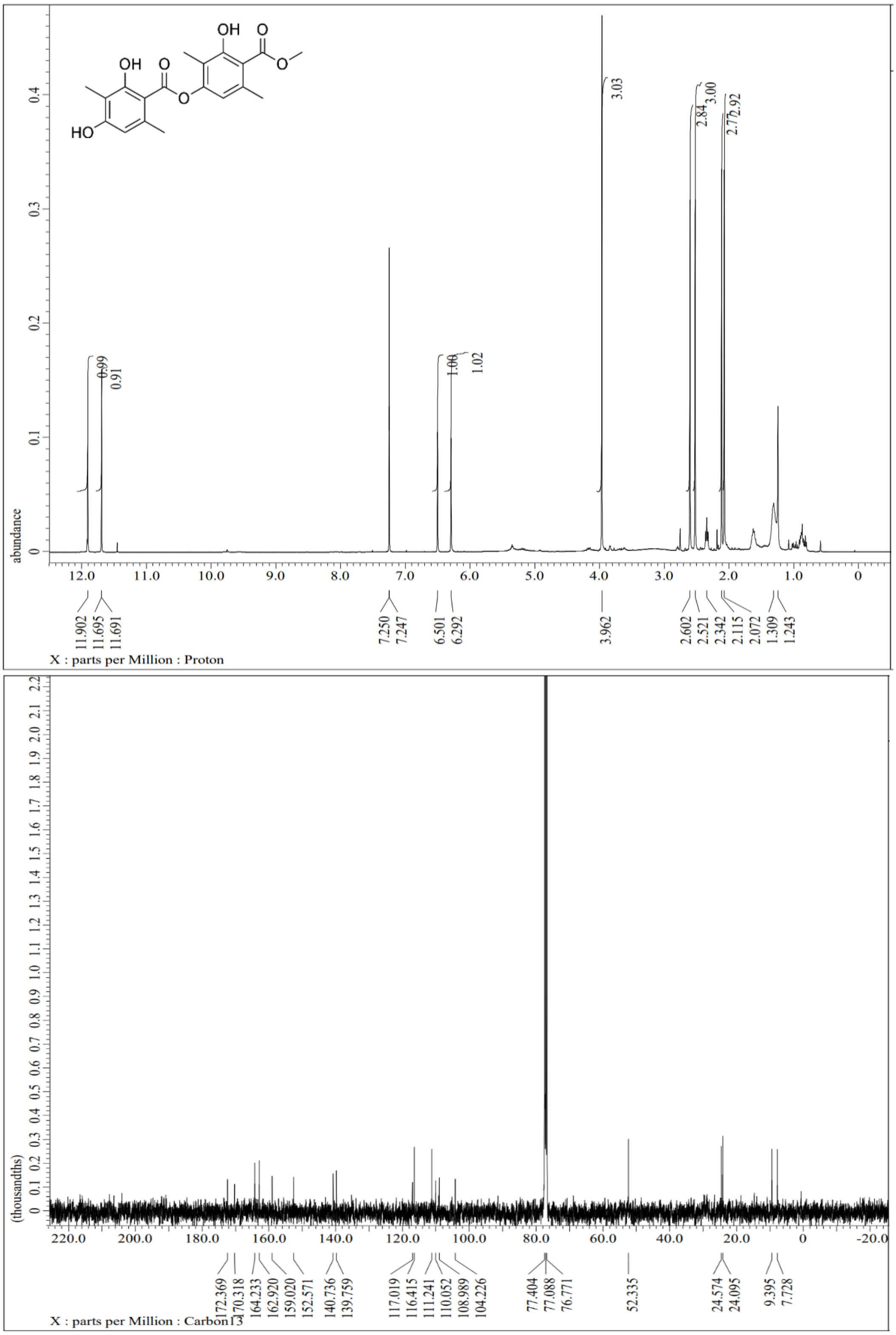

Fig. S4B. NMR analysis for proatranorin I. ^{1}H-NMR (400 MHz; upper panel) and ^{13}C-NMR (100 MHz; lower panel) spectra of **2** in $CDCl_3$.

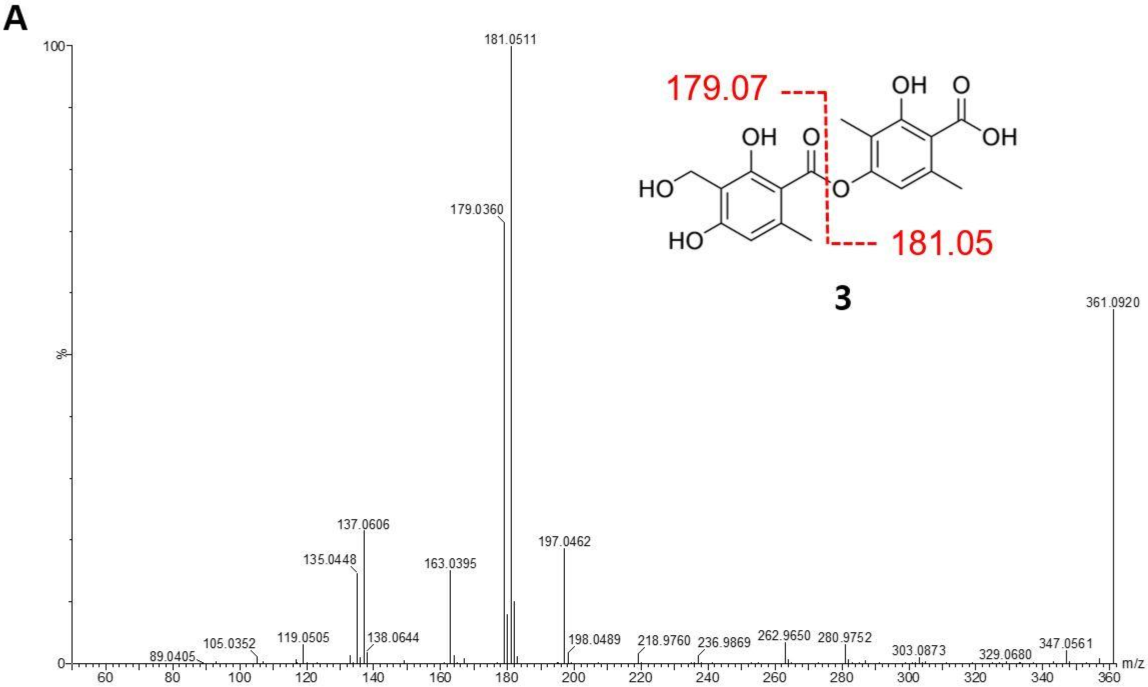

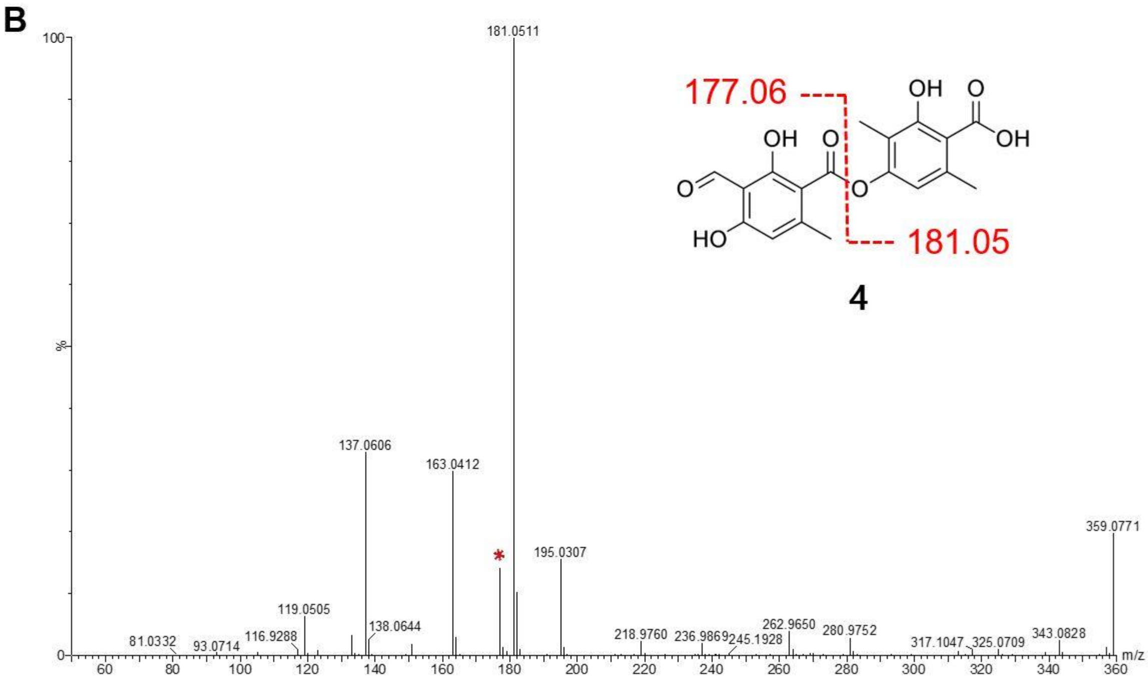

Fig. S4C. LC–MS/MS analysis for proatranorins II and III. The upper MS/MS spectrum indicates a fragmentation mechanism of compound **3**. The lower MS/MS spectrum indicates afragmentation mechanism of compound **4**. Red asterisk indicates a peak corresponding to *m/z* 177.06.

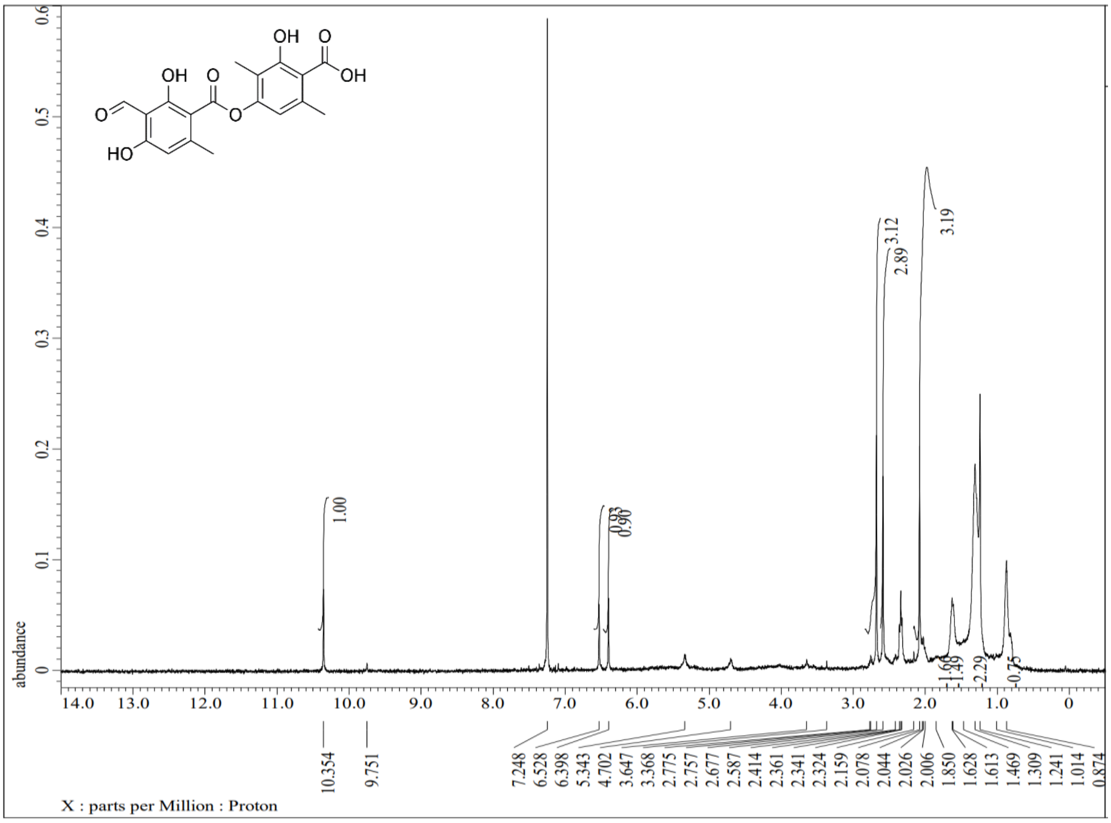

Fig. S4D. NMR analysis for proatranorin III. ^{1}H-NMR (400 MHz) spectrum of **4** in $CDCl_3$.

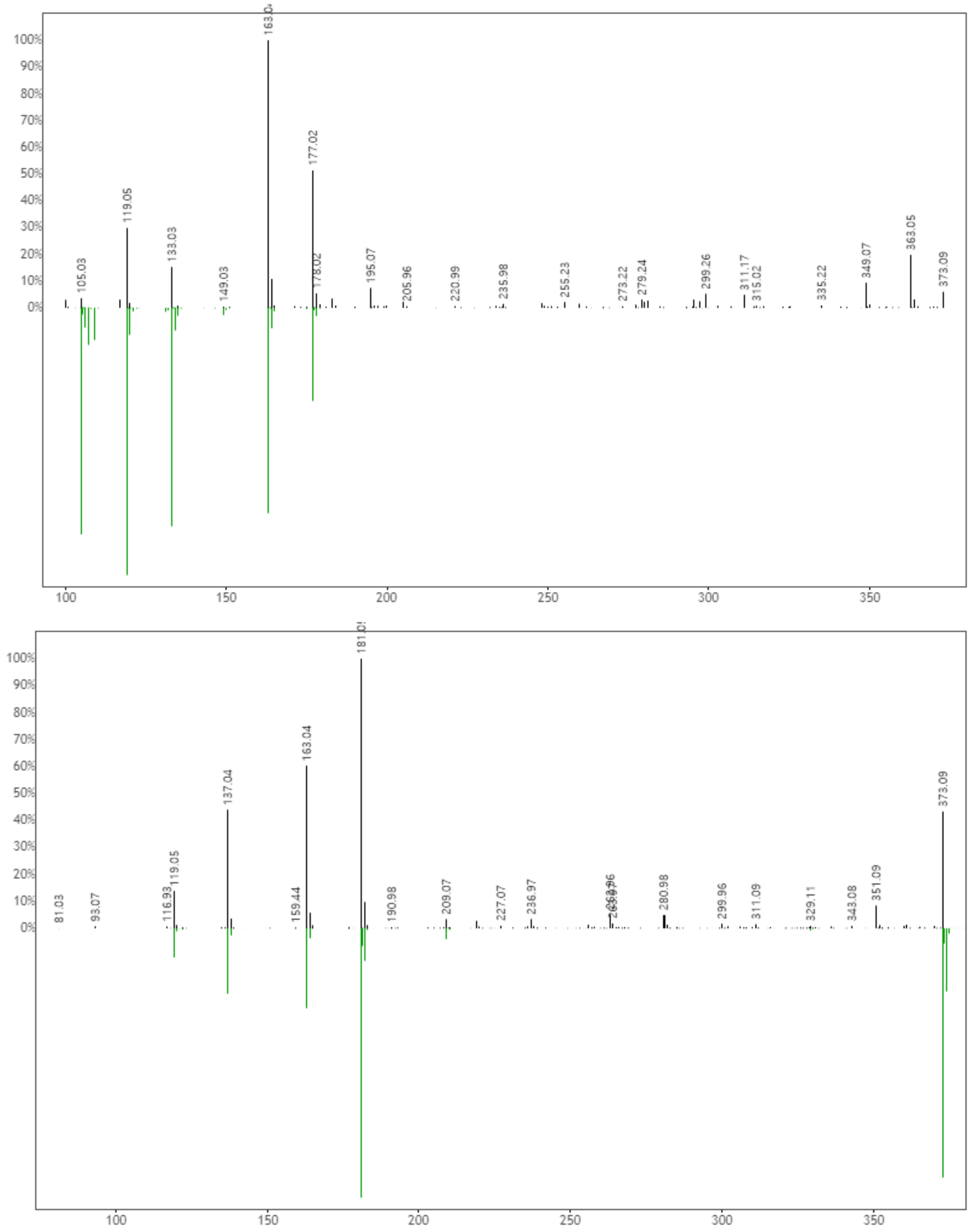

Fig. S4E. MS/MS spectral matching. The upper panel shows MS/MS spectrum of compound **5** observed in a strain expressing the *atr1*, *atr2* and *atr3* (black) and the reference spectrum of atranorin (green) in the Lichen Database (green). The lower panel shows MS/MS spectrum of compound **6** (black) and the reference spectrum of baeomycesic acid (green).
