# Supplementary material for: Linking a Gene Cluster to Atranorin, a Major Cortical Substance of Lichens, through Genetic Dereplication and Heterologous Expression
Source: mBio. 2021 Jun 22;12(3):e01111-21. doi: 10.1128/mBio.01111-21 (PMC8262933; doi:10.1128/mBio.01111-21)
Supplement: FIG S5 [file mbio.01111-21-sf005.pdf]

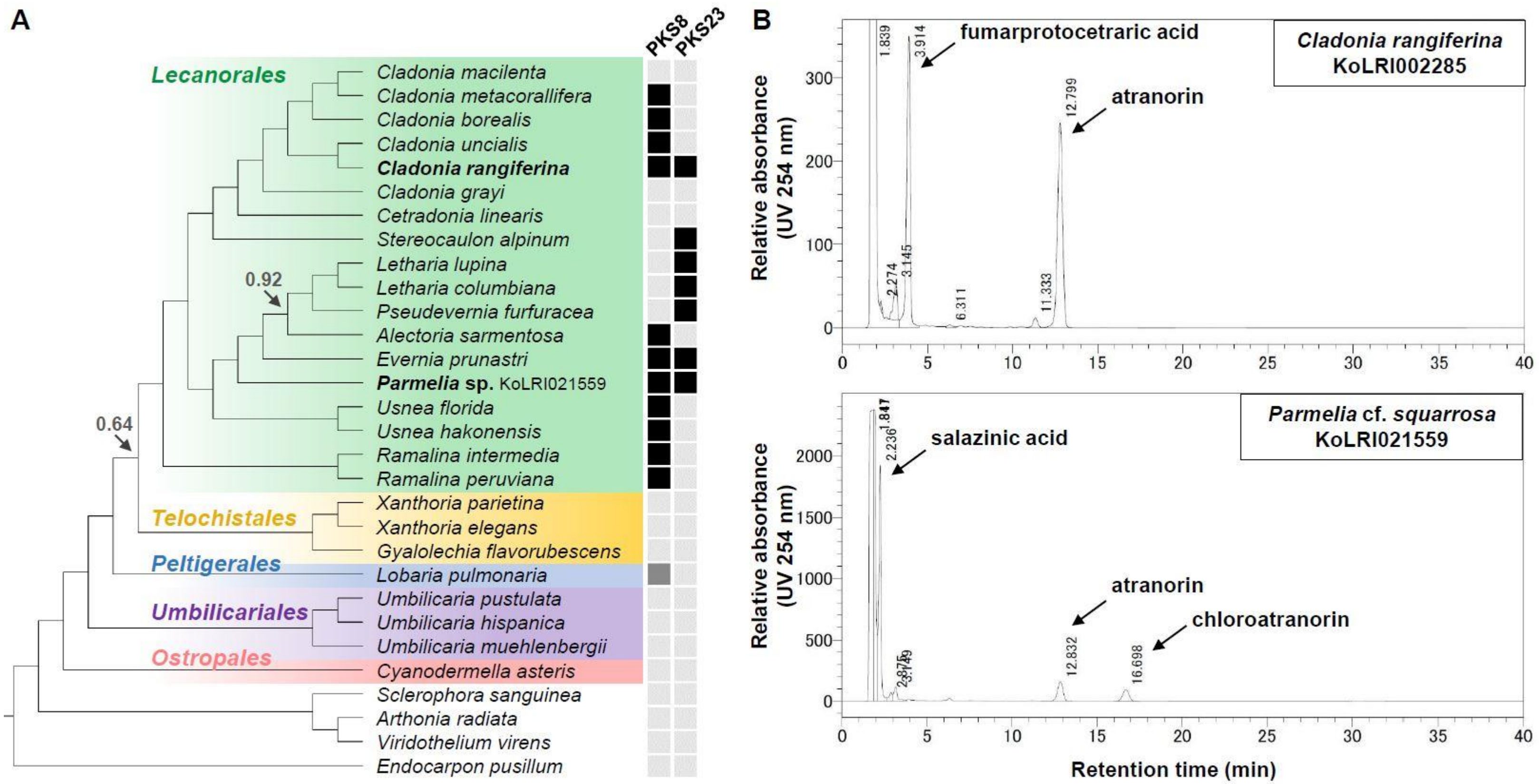

Figs. S5A and S5B. Lichens with atranorin and usnic acid BGCs (**A**) Phyletic distribution of PKS8 family (*aka*. MPAS) and PKS23 (Atr1) family that is responsible for biosynthesis of usnic acid and atranorin, respectively. Black squares indicate the presence of indicated PKS families, and a grey square for *Lobaria pulmonaria* indicates a partial PKS8 gene. Note that *Cladonia rangiferina*, *Evernia prunastri*, and *Parmelia* sp. KoLRI021559 possess both PKS8 and PKS23 BGCs. (**B**) HPLC profiles of acetone extracts of *C. rangiferina* (upper panel) and *Parmelia* sp. KoLRI021559 (lower panel). Lichen substances are identified by comparison their retention time and UV spectra to the lichen substance database. Note that *C. rangiferina* and *Parmelia* sp. produce atranorin as a cortical substance and a 3MOA-derived depsidone as a medullary substance (fumarprotocetraric acid for *C. rangiferina*; salazinic acid for *Parmelia* sp.), lacking usnic acid.

C

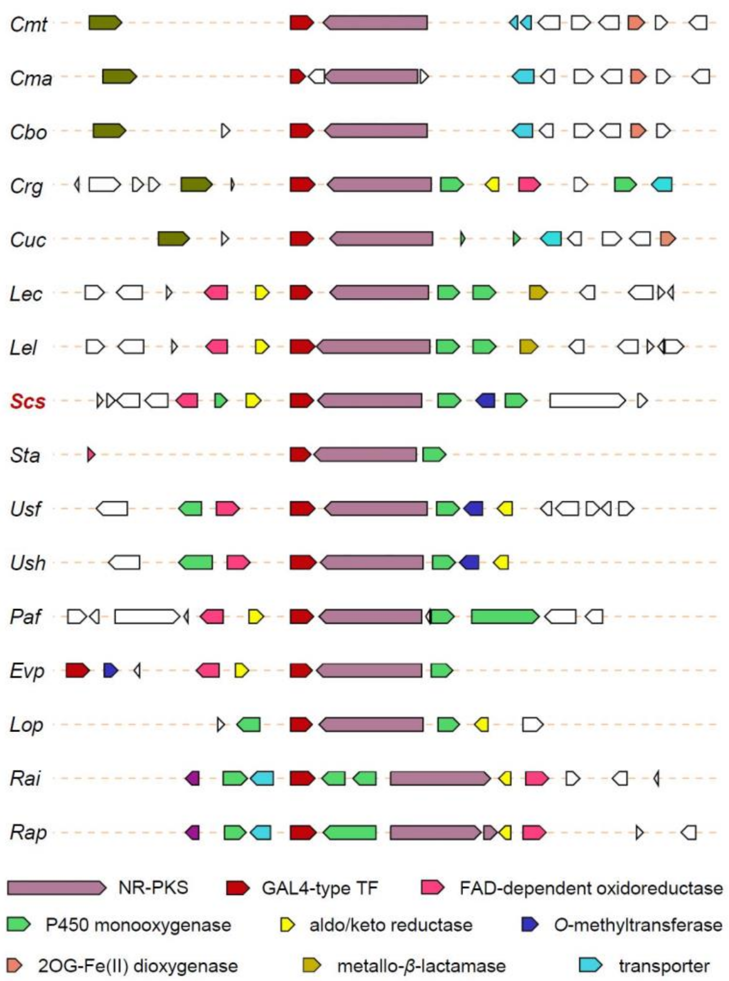

Fig. S5C. Detection of conserved PKS1 BGCs in lichens. Sixteen BGCs including a PKS1 homolog are identified from 1,527 BGCs detected in the 30 genomes, using the CORASON analysis pipeline. A GAL4-type transcription factor and the PKS1 BGC in *C. rangiferina* (*Crg*) are used as the query gene and as the query gene cluster, respectively. Lichen species with a homologous PKS1 BGC are labelled with their abbreviated names, as in Figure 1. Note that *Sclerophora sanguinea* (*Scs*, labelled in red) that belongs to Coniocybomycetes possesses a homologous PKS1 BGC. Genes related to secondary metabolism are color-coded, based on their predicted function. NR-PKS, non-reducing type I PKS; TF, transcription factor.

D

	NR-PKS groups										
	I	II	III	IV	V	VI	VII	VIII	IX	NC	Sum
C. borealis	1	4	1	3	2	4	1	2	2	1	21
C. grayi	3	3	0	1	0	0	0	0	2	1	10
C. macilenta	2	3	2	3	2	2	1	2	2	0	19
C. metacorallifera	2	3	1	3	1	2	1	2	2	0	17
C. rangiferina	3	3	1	1	1	1	0	1	3	1	15
C. uncialis	1	3	1	0	2	2	1	2	2	2	16
S. alpinum	2	1	0	0	0	1	0	1	3	0	8
A. fumigatus	0	0	1	0	3	1	1	1	0	0	7
A. nidulans	1	0	1	1	3	3	6	0	0	0	15
A. niger	0	1	1	0	2	1	3	0	0	1	9
A. terreus	0	1	0	1	1	2	3	0	0	0	8

Fig. S5D. NR-PKS diversity in *Cladonia* and *Aspergillus* species. NR-PKSs in the six *Cladonia* spp., *Stereocaulon alpinum* and four *Aspergillus* spp. are categorized by the nine phylogenetic groups (Groups I–IX). The information on NR-PKSs in the four *Aspergillus* spp. are retrieved from Romsdahl and Wang 2019. The PKS16 family is considered to belong to the Group I, although the PKS16 formed a monophyletic clade sister to the Group I (also see Fig. 5). NC, not categorized.
