# Supplementary material for: Linking a Gene Cluster to Atranorin, a Major Cortical Substance of Lichens, through Genetic Dereplication and Heterologous Expression
Source: mBio. 2021 Jun 22;12(3):e01111-21. doi: 10.1128/mBio.01111-21 (PMC8262933; doi:10.1128/mBio.01111-21)
Supplement: TABLE S2 [file mbio.01111-21-st002.docx]

Table S2. Primers used in this study.

| **Primers** | **Sequence (5'-3')** | **Description** |
| --- | --- | --- |
| Sol1PpDS33_fwd | AGATCTCGGCACGTTTTGATCGCAAAG | *sol1* promoter cloning into pDS33 |
| Sol1PpDS33_rev | AAGCTTAGTAATATCTGGCGATGAAGCGTCAG | *sol1* promoter cloning into pDS33 |
| Inf_Sta046440_fwd | CAGATATTACTAAGCTCATGGCGTCTAACCATGAGGAG | PKS23 cloning into pDS35 |
| Inf_Sta046440_rev | GTAACGTTAAGTGGATCCTTACAACTAAAGCTCTCAATCAACCC | PKS23 cloning into pDS35 |
| Sta046440_check1F | GGACGATGAGAACGTCCTAG | PKS23 sequencing |
| Sta046440_check2F | CGAGTCAGGTGATCCAGTG | PKS23 sequencing |
| Sta046440_check3F | CCAATCATACTGAGCGCTG | PKS23 sequencing |
| Sta046440_check4F | CACATGTCTGTGGCTTGAG | PKS23 sequencing |
| Sta046440_check5F | CAACTCCGGTGATGATTG | PKS23 sequencing |
| Sta046440_check6F | CAACCCTTCCACATATTGAG | PKS23 sequencing |
| pII99_sol5_fwd | GAATTCAGAGCGACAGTGAAAGC | *sol5* promoter cloning into pII99 |
| pII99_sol5_rev | AGATCTA**GCTAGC**TTGGCGTGATGTCAGTCAATGCTG | *sol5* promoter cloning into pII99 (NheI enzyme site in bold) |
| pII99_tef1a_fwd | GAATTCCAGCCGAGACAGCAGAATCAC | *tef1α* promoter cloning into pII99 |
| pII99_tef1a_rev | AGATCTA**GCTAGC**GTGTTATGTTTTGTGGAATATAAAAGGG | *tef1α* promoter cloning into pII99 (NheI enzyme site in bold) |
| Inf95_Sta04642_fwd | ATCACGCCAAGCTAGCACGATGACTTCCGTTGATACAATG | *atr3* cloning into pII95 |
| Inf98_Sta04642_fwd | AAACATAACACGCTAGCACGATGACTTCCGTTGATACAATG | *atr3* cloning into pII98 |
| Inf99_Sta04642_rev | GGAGACCGGCAGATCCCGTCACATGTCCGTCATAATAAC | *atr3* cloning into pII95 and pII98 |
| Inf98_Sta04643_fwd | AAACATAACACGCTAGAACCATGGCTCTCCTAGACACAATTG | *atr2* cloning into pII95 |
| Inf95_Sta04643_fwd | ATCACGCCAAGCTAGAACCATGGCTCTCCTAGACACAATTG | *atr2* cloning into pII98 |
| Inf99_Sta04643_rev | GGAGACCGGCAGATCGAGGAGCAAAGGAACAAGGAACAG | *atr2* cloning into pII95 and pII98 |
| pII99_MK5 | CGTCAAGAGACCTACGAGACTG | Replacing *nptII* with *ble* in the plasmid *tef1α*::*atr2*/pII98 |
| pII99_MK3 | GCTATACTTCTAGGTCTTGGAAGAG | Replacing *nptII* with *ble* in the plasmid *tef1α*::*atr2*/pII98 |
| Inf_BLE_pII99_fwd | GTAGGTCTCTTGACGCATTAAGACCTCAGCGCTAGTGG | Replacing *nptII* with *ble* in the plasmid *tef1α*::*atr2*/pII98 |
| Inf_BLE_pII99_rev | GACCTAGAAGTATAGCGGTGTTACGGAGCATTCACTAGG | Replacing *nptII* with *ble* in the plasmid *tef1α*::*atr2*/pII98 |
| L5 | GAGGTCTTCACACCACAAGTTCG | Generating “clean host” |
| L3 | GGCAAAGGAATAGAGTAGATGCCGACCGGGCTCTTGCGTAGTGGTACAGATG | Generating “clean host” |
| R5 | GTTGACCTCCACTAGCTCCAGCCAAGCGAATAGGTAACAAAGCCAGCCGAG | Generating “clean host” |
| R3 | GAGAATTGCGGCGCAGGATGTTC | Generating “clean host” |
| N5 | CTGACTTTCTTCTTGCAGCCCTGC | Generating “clean host” |
| N3 | CTAACGATGTCGTTAGTCGCCTTG | Generating “clean host” |
| HYG-F | GCTTGGCTGGAGCTAGTGGAG | Generating “clean host” |
| HYG-R | CGGTCGGCATCTACTCTATTCCTT | Generating “clean host” |
| YG-F | CGATGTAGGAGGGCGTGGATATGTC | Generating “clean host” |
| HY-R | TGTAGTGTATTGACCGATTCCTTGCG | Generating “clean host” |
| P1_fwd | GGACGATGAGAACGTCCTAG | RT-PCR analysis |
| P1_rev | GAGAGTTCCTCATGCTCGTC | RT-PCR analysis |
| P2_fwd | CATCGACGACACGGAGATACTG | RT-PCR analysis |
| P2_rev | GACCGTGAGATTCTTTGAAGAGG | RT-PCR analysis |
| Actin1_fwd | CAATGGTTCGGGTATGTGCAAG | RT-PCR analysis |
| Actin1_rev | GAAGAGCGAAACCCTCGTAGAT | RT-PCR analysis |
